# La–[^18/19^F]fluoride complexes: a novel addition to the radiofluorination family

**DOI:** 10.1039/d5dt02645h

**Published:** 2026-03-30

**Authors:** Martin Behe, Stefan Gruber, Cen Li, Georg Schreckenbach, Linjing Mu, Margret Schottelius, Radmila Faizova

**Affiliations:** a Center for Radiopharmaceutical Sciences, Paul Scherrer Institute 5232 Villigen PSI Switzerland; b Department of Chemistry and Applied Biosciences, ETH Zürich Vladimir-Prelog-Weg 1–5 8093 Zürich Switzerland; c Department of Chemistry, University of Manitoba Winnipeg Manitoba R3T 2N2 Canada; d Translational Radiopharmaceutical Sciences, Departments of Nuclear Medicine and Oncology, Centre Hospitalier Universitaire Vaudois (CHUV) and University of Lausanne (UNIL) Rue du Bugnon 25A Agora CH-1011 Lausanne Switzerland

## Abstract

Here, we report the synthesis and evaluation of La–[^18/19^F]fluoride coordination complexes with macrocyclic ligands, expanding the family of metal–[^18^F]fluoride coordination species for PET imaging applications. Fluorination of a series of La complexes with macrocyclic chelators (L = DOTA, DOTAM, macropa, and DOTpy) was explored, and the resulting products were characterized using ^19^F and ^1^H NMR, mass spectrometry, and X-ray spectroscopy. The stability of these complexes in water was found to inversely correlate with the electron-donating properties of the chelator. Notably, [LaF(L)]^*x*^ complexes with macropa and DOTpy exhibited high stability in organic solution, with [LaF(DOTpy)]OTf_2_ also demonstrating stability in water. DFT study (PBE-D3(BJ)/TZ2P, COSMO) indicates that the ΔΔ*G* magnitudes between [LaFL] and [La(H_2_O)L] could explain these results: much higher for DOTpy (21.66 kcal mol^−1^) and DOTAM (15.86 kcal mol^−1^) than for DOTA (6.78 kcal mol^−1^) and Macropa (8.05 kcal mol^−1^) supports the observed stabilities. Radiofluorination of [LaF(DOTpy)]OTf_2_ afforded [^18^F][LaF(DOTpy)]^2+^ in high radiochemical purity, representing the first example of a La–[^18^F]fluoride coordination complex. These findings expand the scope of metal–[^18^F]fluoride coordination chemistry and lay the groundwork for developing La/Ac-based fluoride complexes as theranostic pairs, with applications in ^225^Ac-based radiopharmaceuticals for personalized dosimetry and PET-guided therapy monitoring.

## Abstract 2

Novel La–[^18/19^F]fluoride complexes lay a foundation towards ^18^F/^225^Ac/La theranostics. Systematic evaluation of La–F coordination chemistry reveals a fundamental trade-off: ligand electronics govern La–F bond strength and stability.

## Graphical abstract

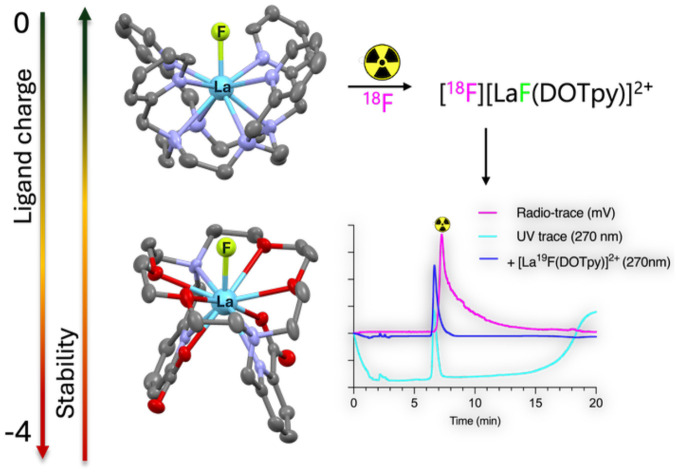

## 1. Introduction

Theranostics, the integration of diagnostic imaging and targeted therapy, has emerged as a transformative approach in personalized medicine.^1,2^ By combining molecular imaging with therapeutic interventions, theranostics enables precise diagnosis, real-time monitoring, and tailored treatment strategies. The clinical success of radiotheranostic agents such as [^68^Ga]Ga-DOTATATE and [^177^Lu]Lu-DOTATATE for neuroendocrine tumors (NETs),^3,4^ as well as [^68^Ga]Ga-PSMA-11 and [^177^Lu]Lu-PSMA-617 for prostate cancer^5,6^ has demonstrated the potential of this approach to revolutionize nuclear medicine and enhance precision oncology. Building on these successes, targeted alpha therapy (TAT) offers a promising next-generation strategy for cancer treatment.

Actinium-225 (^225^Ac) is a promising candidate for TAT because of its relatively long half-life and high-energy alpha particle emission.^7–9^ However, despite promising results in early studies, its clinical use is currently limited. Since α-emissions cannot be directly visualized using conventional nuclear medicine imaging methods, quantitative imaging and dosimetry for TAT pose major challenges. This, in turn, limits personalization of dosing regimens and complicates the establishment of reliable dose–response relationships. Furthermore, the fundamental chemistry of actinium is underdeveloped due to its radioactivity, low availability, and challenges associated with handling this element. Therefore, important chemical properties of Ac-complexes are still only partially understood. To address these challenges, La is often used as a surrogate for Ac because of their similar ionic radii, enabling the investigation of actinium's chemical behavior without the associated difficulties.^10^

Among the radionuclides used in theranostics, fluorine-18 holds a unique position as a preferred positron emitter due to its favorable physical and chemical properties.^11^ With a half-life of 109.8 minutes, high positron emission efficiency (97%), and a low positron energy (maximum 0.635 MeV), fluorine-18 enables high-resolution PET imaging while allowing for centralized production and distribution to satellite facilities.

Metal–fluoride coordination complexes represent a promising class of ^18^F-labeled compounds for theranostic applications. These complexes offer straightforward radiosynthesis methods and tunable properties based on the choice of metal ion and ligand system.

McBride and colleagues pioneered a novel radiofluorination method in 2009, using [^18^F]fluoride capture by a metal chelate in water *via* the Al[^18^F]F system.^12^ This approach leveraged coordination chemistry principles to facilitate fluoride incorporation. While ^18^F-coordination chemistry has been successfully demonstrated with some Group 3,^13–15^ Group 13^16,17^ and transition metals,^18^ lanthanide (Ln)-based systems remain largely underexplored. Only one unsuccessful ^19^F-fluorination attempt of [LaCl_3_ (Me_3_-tacn)(OH_2_)] (Me_3_-tacn = 1,4,7-trimethyl-1,4,7-triazacyclononane) with [NMe_4_]F in CH_3_CN has been reported, resulting in decomposition of the complex and the formation of LaF_3_ polymer.^15^

[LnF(L)]^*x*^ complexes have been previously studied for their luminescence,^19^ fluoride sensing,^20,21^ and magnetic properties, particularly in single-molecule magnets (SMMs).^22^ The fluoride ligand plays a crucial role in creating a strong axial crystal field that enhances magnetic anisotropy in lanthanide ions such as Ce(iii), Nd(iii), Dy(iii), and Tb(iii).^23,24^ Lanthanum–fluoride coordination was also explored due to their applications in catalysis^22,25^ (*e.g.* C–F bond activation), spectroscopy, and fluoride sensing (La–alizarin complexone (ALC) system with fluoride; photometric and potentiometric studies). Several studies have demonstrated that the nature and structure of the coordinating ligands significantly influence the fluoride affinity of these complexes.^24^

In this study, we report the synthesis and evaluation of La–^18/19^F fluoride complexes as potential novel imaging agent. By exploring various macrocyclic chelators, a combination of computational, multi-nuclear spectroscopic, mass spectrometry, X-ray crystallography, and radiochemical methods, we aim to establish structure–stability relationships that inform the design of robust La–^18/19^F fluoride complexes. This work not only expands the family of metal–^18^F fluoride coordination species, but also lays the foundation for future applications in radiotheranostics, including targeted alpha therapy with [^225^Ac].

## 2. Experimental

Synthetic procedures and characterisation details are presented in the SI.

### 2.1. ^19^F-fluorination of selected [La(L)]X_0–3_

The reaction of La(OTf)_3_ with the selected ligands in MeOH or aqueous media afforded complexes 1–4 which were characterized by ^1^H NMR and MS, and have a general formula [La(L)X]X_0–3_. Subsequently, the *in situ*^19^F-fluorination of the resulting La complexes was investigated both in organic and aqueous media with TBAF. Notably, the addition of TBAF was always performed dropwise at room temperature to prevent the precipitation of LaF_3_. In the absence of a chelator immediate formation of insoluble LaF_3_ was observed, accompanied by the disappearance of the ^19^F NMR resonance (Fig. S1).^26^ All reactions were monitored by ^19^F NMR spectroscopy and mass spectrometry. Upon identification of a novel fluorinated species, crystallization was attempted, yielding several samples suitable for X-ray diffraction analysis. Following confirmation of La–F bond formation, the novel complexes were fully characterized by quantitative ^19^F NMR, elemental analysis, high-resolution mass spectrometry (HR-MS), and X-ray crystallography.

In a typical procedure in MeOH, if required, a deprotonated solution of the ligand (1.1 equiv.) in methanol (1 mL) was prepared by the addition of an equimolar amount of Et_3_N. A colorless solution of lanthanum triflate (1 equiv.) in methanol (0.5 mL) was then added dropwise to the ligand solution. The resulting mixture was stirred at ambient temperature or 60 °C for 1–24 hours. The formation of the starting complex was confirmed by LC-MS. After cooling to ambient temperature, a colorless solution of TBAF (1–3 equiv.) was added dropwise, and the mixture was stirred for 30 minutes. LC-MS and the ^19^F NMR spectra were subsequently recorded to confirm the identity of the fluorinated product.

In a typical procedure in aqueous buffer, to a solution of the ligand (1.1 equiv.) in NH_4_OAc (pH ≈ 5) (1 mL), a colorless solution of lanthanum triflate (1 equiv.) in NH_4_OAc (pH ≈ 5) (0.5 mL) was added dropwise. The resulting mixture was stirred at ambient temperature or 80 °C for 1–24 hours. The formation of the starting complex was confirmed by LC-MS. After being cooled to ambient temperature, a colorless solution of TBAF (1–7 equiv.) was added dropwise to the mixture and stirred for 30 minutes. LC-MS and the ^19^F NMR spectra were subsequently recorded to confirm the identity of the fluorinated product.

### 2.2. ^18^F-fluorination of [LaF(DOTpy)]^2+^

Anhydrous, no-carrier added [^18^F]fluoride was obtained as [^18^F]BnEt_3_NF in methanol solution using a previously published QMA processing procedure.^27^

To 100 μL of 10 mM solution of lanthanum triflate in MeOH 100 μL of [^18^F]BnEt_3_NF (50–100 MBq) in methanol were added. The reaction mixture was left at 60 °C for 30 minutes. Then, 20 μL of a 100 mM stock solution of DOTpy ligand in methanol were added to the reaction mixture. The reaction mixture was left at 60 °C for 5 minutes and analyzed by radioHPLC. The identity of the radiolabeled complex was further confirmed by co-injection of a large excess of non-radioactive reference complex 8, [LaF(DOTpy)]OTf_2_, into the radiolabeling mixture, followed by radioHPLC analysis.

### 2.3. Computational methods

To get further insight into the formation and stability of [LaX(L)] (X = F^−^OH^−^ and H_2_O) complexes, a Density Functional Theory (DFT) study was conducted using the Amsterdam Density Functional program (ADF 2021).^28,29^ The structures of the La complexes were optimized using the PBE-D3(BJ) functional,^30–32^ a scalar relativistic ZORA Hamiltonian,^33–36^ and triple-ζ plus two polarization functions (TZ2P) Slater-type basis sets with the frozen core approximation using a small core. Frequency calculations were performed to determine thermodynamic properties (Gibbs free energy at 298 K) of each structure and to verify the true minima on the potential energy surface. All calculations were carried out under aqueous solution conditions using the conductor-like screening solvation model (COSMO).^37–40^

For calculations of thermodynamics of water molecules in water, an effective pressure of *p* = 1354 atm, which was obtained from *p* = *ρ*_w_RT, corresponding to the experimental density of liquid water *ρ*_w_ = 997.02 kg m^−3^ at 298 K,^41^ was used to mimic the condensed phase to model translational degrees of freedom in the solvent for an entropy correction.

Energy Decomposition Analysis (EDA)^42^ was performed to obtain further insight into the bonding between La^3+^ and the axial ligands (F^−^OH^−^ and H_2_O) using LaL complexes and the axial ligands as interacting fragments. Charge transfer analysis (Voronoi deformation density (VDD)^43^ and Hirshfeld^44^ charge analysis methods) was also carried out.

## 3. Results and discussion

### 3.1. Ligand choice and the outline of chemical synthesis

In this study, we investigated how ligand charge, denticity, and donor group basicity affect fluoride binding to lanthanum(iii), used here as a non-radioactive surrogate for actinium. Therefore, we selected previously reported chelators DOTA, DOTAM, Macropa, and DOTpy, (Table 1) known for their ability to form stable complexes with large trivalent cations, including La. The stability of these complexes is crucial for creating coordination environments allowing for subsequent fluorination. By comparing ligands with differing structural and electronic properties, we aimed to systematically assess how coordination geometry and ligand design influence both the stability and reactivity of the resulting La–F containing complexes.

**Table 1:** Structures and abbreviated names of the ligands discussed and investigated in this article

	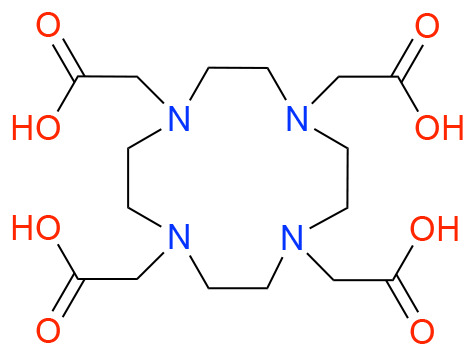	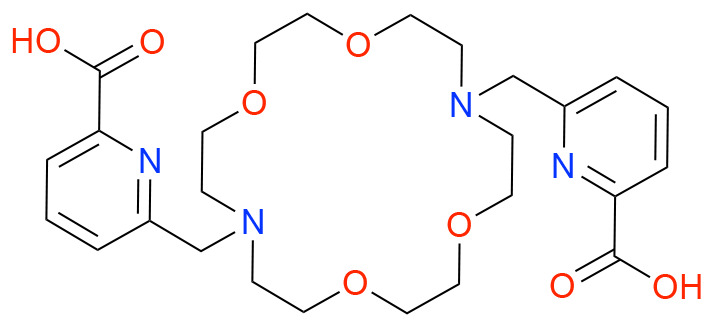	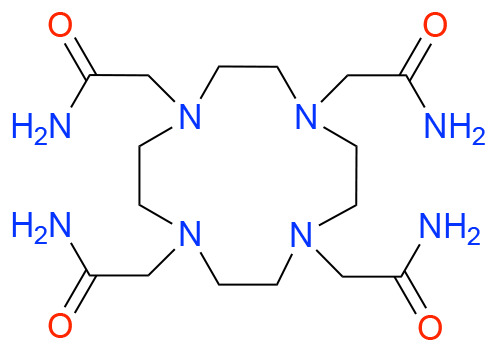	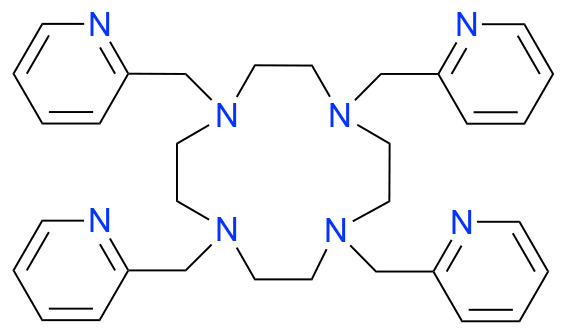
Charge	−4	−2	0	0
Donor sphere	N_4_O_4_	N_4_O_6_	N_4_O_4_	N_8_
La complex	[La(HDOTA)] (1)	[La(macropa)]OTf (2)	[La(DOTAM)(OTf)]OTf_2_ (3)	[La(DOTpy)(OTf)]OTf_2_ (4)

The ^19^F-fluorination of the *in situ* prepared [La(DOTA)]^−^ in methanol and [La(HDOTA)] in aqueous NH_4_OAc (pH ≈ 5) using an excess of TBAF did not yield any detectable formation of fluoride-containing species. This lack of fluorination occurred despite prolonged reaction times and elevated temperatures (Fig. S2 and S3).

**Fig. 1:**
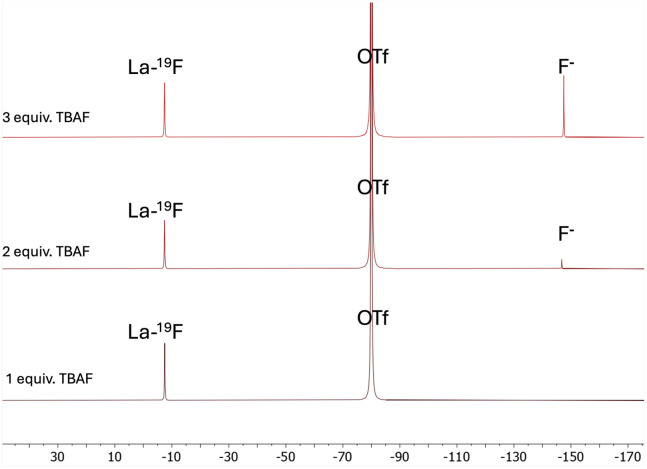
^19^F NMR spectrum (376 MHz, MeOD, 298 K) after the addition of 1 equiv. of TBAF (bottom); 2 equiv. of TBAF (middle); 3 equiv. of TBAF (top) to [La(macropa)]^+^.

To assess the influence of ligand charge on fluoride binding affinity, the macropa ligand was chosen due to its reported efficiency in complexing large metal cations, including La^3+^.^10^ Following the *in situ* synthesis of [La(macropa)]^+^ in methanol, the addition of 1–3 equiv. of TBAF in MeOH was performed and monitored by ^19^F NMR. The NMR spectrum showed a resonance corresponding to triflate (*δ* = −80 ppm), and a new resonance at *δ* = −7 ppm, assigned to La–F bound fluoride. The resonance at −148 ppm, observed upon the addition of the second equivalent of TBAF, indicates the presence of free fluoride, suggesting a 1 : 1 stoichiometry between La and fluoride in this complex (Fig. 1, Fig. S4).

**Fig. 2:**
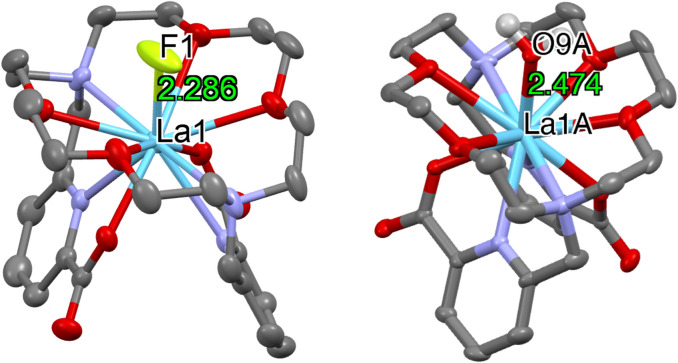
Ellipsoid plot at 50% probability of complex 5 (left) and complex cation 6 (right). Most hydrogen atoms have been omitted for clarity. The non-coordinated triflate ligand in 6 has also been removed (C are represented in gray, O in red, N in dark blue, La in blue, and F in green).

The novel fluorinated complex 5 was prepared in methanol solution in near-quantitative yield, as determined by ^19^F NMR spectroscopy (Fig. S4). Upon crystallization of the reaction mixture, by vapor diffusion of Et_2_O, colorless crystals of 5 suitable for X-ray diffraction were obtained. The solid-state structure of [LaF(macropa)] (5) was determined by X-ray diffraction, showing the presence of the La–F bond (2.286(5) Å) (Fig. 2).

Further, the stability of the resulting complex in methanol solution was evaluated over two weeks by monitoring with ^19^F NMR spectroscopy. During this time, increasing amounts of insoluble LaF_3_ were observed, indicating a gradual degradation of the fluorinated complex. Subsequently, the stability of the resulting [LaF(macropa)] complex in water was examined. Upon addition of 300 μL (50% by volume) of aqueous HEPES solution (pH = 7), a complete disappearance of the resonance assigned to a La–F bond was observed (Fig. S5). This behavior was attributed to competitive binding between water molecules and fluoride anions at the La center, leading to the displacement of fluoride and formation of a hydrated complex, [La(H_2_O)(macropa)]OTf, as confirmed by the crystal structure obtained from the reaction mixture (Fig. 2). An attempt to synthesize complex 5 directly in aqueous media was unsuccessful (Fig. S6), further confirming the instability of complex 5 in the presence of water.

X-ray crystal structures of complexes 5 and 6 demonstrate similar coordination geometries, forming 11-coordinate complexes with the macropa ligand. In both cases, the two picolinate arms are positioned on the same side of the macrocycle, while a fluoride ligand (5) or an inner-sphere water molecule (6) occupies a position on the opposite side of the 18-membered ring. Overall, structural parameters are similar to those previously reported [La(Hmacropa)(H_2_O)]·(ClO_4_)_2_ ^10^ and can be found in Table S4.

To evaluate whether further reducing the ligand charge could enhance the stability of fluorinated La complexes, the neutral amide derivative of DOTA, namely DOTAM, was investigated. Upon reaction with an equimolar amount of fluoride, both in methanol (Fig. S7) and aqueous solutions (Fig. S8), the formation of a new species was observed by mass spectrometry and ^19^F NMR spectroscopy. A new resonance in the ^19^F NMR spectrum was observed at −13.7 ppm, which was assigned to [LaF(DOTAM)]^2+^ (7) species. This chemical shift is consistent with La-bound fluoride, reflecting the increased deshielding compared to free fluoride anion (−122 ppm). Further addition of fluoride source resulted in the increased formation of insoluble LaF_3_. The formation of a precipitate was also observed upon longer reaction times and increased reaction temperatures. Multiple crystallization attempts did not yield crystals of the desired product.

**Fig. 3:**
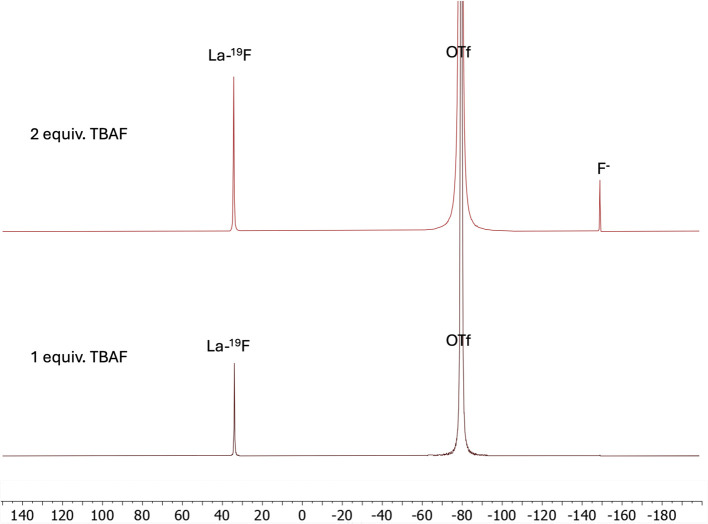
^19^F NMR spectrum (376 MHz, MeOD, 298 K) 1 hour after the addition of 1 equiv. (bottom) and 2 equiv. of TBAF (top) to [La(DOTpy)(OTf)]^2+^ (4).

Finally, another neutral chelator, DOTpy, was investigated. This ligand has previously been shown to form stable complexes with La^45^ and has been used in the synthesis of various fluorinated lanthanide complexes.^23,24^ However, fluorination had not been previously reported for the La analog. To address this, the synthesis of [LaF(DOTpy)]^2+^ was first attempted in methanol using a standard procedure. ^19^F NMR spectrum indicated a quantitative conversion to a new fluorine-containing species with a chemical shift of 34 ppm, which was assigned to [LaF(DOTpy)]^2+^. The addition of further equivalents of TBAF resulted in the observation of free fluoride on the ^19^F NMR spectrum (Fig. 3).

**Fig. 4:**
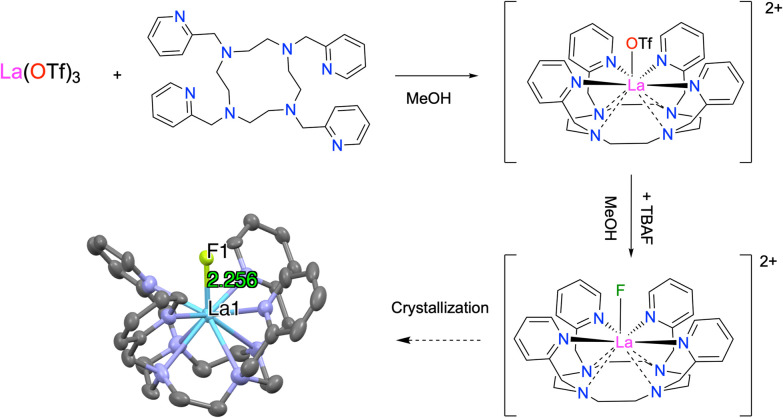
Synthesis and ellipsoid plot at 50% probability of [LaF(DOTpy)]^2+^. The hydrogen atoms have been omitted for clarity. The non-coordinated triflate anions have been removed (C are represented in gray, N in dark blue, La in blue, and F in green).

Upon crystallization of the reaction mixture, by Et_2_O vapor diffusion, colorless crystals of [LaF(DOTpy)]OTf_2_ (8) suitable for X-ray diffraction were obtained (Fig. 4). In the solid-state structure, the La^3+^ ion is coordinated by eight nitrogen donors, four from the aza-crown core and four from pyridine moieties of the ligand's pendant arms, as well as a fluoride in the axial position. Notably, the structure exhibits high symmetry, evident by the asymmetric unit (completed by a two-fold rotation), comprising a half-ligand around the La^3+^ center, a fluoride ligand, and CF_3_SO_3_^−^. The La–F bond in 8 is slightly shorter in comparison to [LaF(macropa)] (5) (2.256(5) Å *vs*. 2.286(5) Å). General structural parameters compare well with the previously reported [LnF(DOTpy)]^2+^.^23^ Complex 8 was obtained in 80% yield and characterized by ^1^H and ^19^F NMR spectroscopy (Fig. S9–S11), HR-MS (Fig. S12), and elemental analysis. Interestingly, ^1^H NMR (Fig. S10) spectrum clearly demonstrates the influence of the axial ligands on the chelate system. ^1^H, ^19^F NMR and HR-MS data confirmed that the La–F bond in complex 8 is stable in methanol for at least 3 weeks.

Having obtained a novel [LaF(DOTpy)]OTf_2_ complex with a highly deshielded fluorine encouraged us to investigate the water stability of complex 8. The ^19^F NMR spectra were recorded in aqueous solutions at pH = 7 and pH = 5 (Fig. S13 and S14), both of which indicated the presence of a resonance assigned to the La–F bond, suggesting the stability of complex 8 in water. Notably, the resonance of La-bound fluoride in water at neutral pH shows a significant upfield shift when compared with the ^19^F NMR spectrum of the methanol solution of the complex 8 (25.1 ppm *vs.* 34 ppm). The stability of the complex 8 in water was evaluated over a period of 3 weeks by ^19^F NMR, demonstrating its persistence in aqueous solution (Fig. S13 and S14).

### 3.2. ^19^F NMR chemical shift analysis

**Fig. 5:**
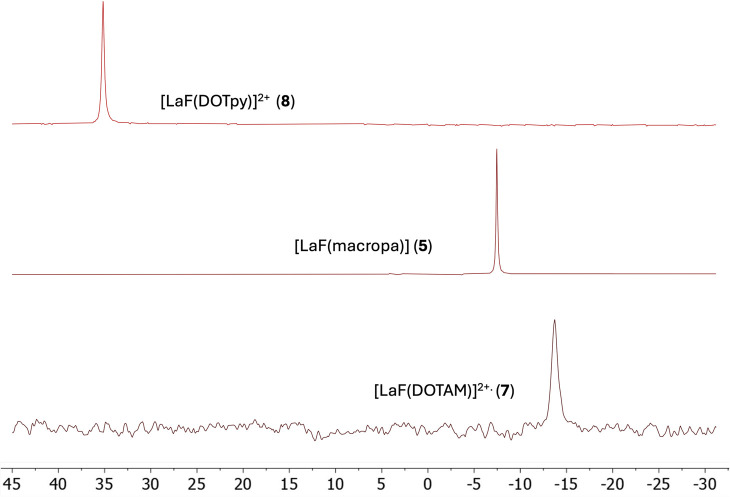
Stacked zoom in on the ^19^F NMR spectra (376 MHz, MeOD, 298 K) of complexes 5, 7, and 8.

To better understand the strength and stability of the La–F bond across the series, an analysis of the corresponding ^19^F NMR spectra was undertaken. As previously noted, the ligand environment significantly influences the chemical shift of the fluoride. Highly deshielded and narrow resonances of complexes 8 and 5 in methanol solutions suggest higher stability of the La–F bond and a rigid conformation in the solution. On the other hand, significant broadening of the [LaF(DOTAM)]^2+^ fluorine resonance indicates the presence of different chemical environments, which could be due to conformational changes or a ligand exchange reaction (Fig. 5).

### 3.3. Density functional theory

#### 3.3.1. Geometries

Following previous work,^46^ To understand the aqueous stability of [LaF(L)] LaL complexes with competing axial ligands (OH^−^, H_2_O) were studied and compared. Structures of LaL and [LaX(L)] (X = F^−^OH^−^H_2_O) were optimized by DFT. The optimized [La(DOTA)]^−^ from previous work^47^ was used for DOTA; DOTAM structures were based on DOTA; Macropa and DOTpy used X-ray structures. In [LaX(macropa)], the axial ligand is opposite the pendant arms, unlike other [LaX(L)]. Optimized [LaF(L)] structures are in Fig. S19; [La(OH)(L)] and [La(H_2_O)(L)] maintain similar conformations, with coordinates in SI. La–X distances (Table S1) follow La–F < La–O(OH^−^) < La–O(H_2_O). The shortest La–F bond is in [LaF(DOTpy)]^2+^, then [LaFDOTAM]^2+^, consistent with their stabilities. Calculated La–X bond lengths in solution compare with crystal structures of [LaF(DOTpy)]^2+^, [LaFmacropa]^0^, and [La(H_2_O)macropa]^+^. For [LaF(DOTpy)]^2+^ and [LaFmacropa]^0^, calculated La–F bonds are shorter (2.216 *vs.* 2.256 Å, 2.239 *vs.* 2.286 Å), while for [La(H_2_O)macropa]^+^, the La–O(H_2_O) bond is longer (2.649 *vs.* 2.502 Å).

#### 3.3.2. Thermodynamics

Stabilities of the LaL and [LaX(L)] (X = F^−^OH^−^ and H_2_O) complexes can be evaluated by the Gibbs free energy of the following designed reactions ((R1) and (R2)).

Complex formation reaction:

R1 [La(H_2_O)_9_]^3+^ + L^*m*−^ + X^*x*−^ → [LaXL]^3−*m*−*x*^ + 9H_2_O

Axial ligand substitution reaction:

R2 [LaX_1_L]^3−*m*−*x*^ + X_2_^*y*−^ → [LaX_2_L]^3−*m*−*y*^ + X_1_^*x*−^

The stabilities of [LaL], [La(H_2_O)L], and [La(OH)L] were compared to [LaFL] *via* ΔΔ*G* values from ligand substitution reaction (R2) (Table S2). Negative ΔΔ*G* indicate [La(OH)L] are slightly more stable than [LaFL] (less than −3.20 kcal mol^−1^), while positive values show [LaL] and [La(H_2_O)L] are less stable, confirming the water stability of [LaFL] for all four ligands. Experimentally, only [LaF(DOTpy)]^2+^ and [LaF(DOTAM)]^2+^ are stable in water; [LaF(DOTA)]^2−^ and [LaF(macropa)] are unstable. The ΔΔ*G* magnitudes between [LaFL] and [La(H_2_O)L] explain these results: much higher for DOTpy (21.66 kcal mol^−1^) and DOTAM (15.86 kcal mol^−1^) than for DOTA (6.78 kcal mol^−1^) and Macropa (8.05 kcal mol^−1^). This smaller stability gap may cause [La(H_2_O)L] dominance in solution when fluoride concentration is low, favoring their formation under standard 1 M F^−^ conditions.

#### 3.3.3. Charge transfer analysis

The partial charges on La and F in the [LaFL] complexes were analyzed using the Hirshfeld and Voronoi charge analysis methods (Table 2). More detailed charge analysis for all complexes can be found in the SI. Both Hirshfeld and Voronoi charge analysis methods resulted in the same trend. The charges on La are smaller than the formal charge +3 while those on F are less negative than the formal charge −1, which indicates that charge was transferred to La and was partially originating from F. Generally, a correlation between the partial charge on F and the stability of [LaFL] complexes can be observed (greater electrostatics → higher stability). The highest (least negative) partial charge on F in [LaF(DOTpy)]^2+^ shows the most charge transfer, which contributes to the highest water stability of the [LaF(DOTpy)]^2+^ complex. This correlation offers a practical design rule: favor neutral, nitrogen-rich donors that maximize electrostatic stabilization of the La–F bond in competitive media.

**Table 2:** Partial charges on La and F by Hirshfeld and VDD methods

Complex	Hirshfeld		VDD	
	La	F	La	F
LaFDOTA^2−^	0.364	−0.493	0.149	−0.509
LaFMacropa	0.432	−0.401	0.171	−0.419
LaFDOTAM^2+^	0.431	−0.448	0.152	−0.445
LaFDOTpy^2+^	0.444	−0.356	0.159	−0.375

#### 3.3.4. Energy decomposition analysis (EDA)

Energy Decomposition Analysis (EDA) was conducted to investigate bonding between La^3+^ and axial ligands (F^−^, OH^−^, H_2_O) in LaL complexes. The intrinsic interaction energy Δ*E*_int_ is decomposed as:Δ*E*_int_ = Δ*E*_pauli_ + Δ*E*_elstat_ + Δ*E*_orb_ + Δ*E*_disp_where Δ*E*_pauli_, Δ*E*_elstat_, Δ*E*_orb_, and Δ*E*_disp_ are the Pauli repulsion, electrostatics, orbital, and dispersion^48^ contributions, respectively.

From Table S3, Δ*E*_elstat_ dominates (61–78%) in most complexes except [LaF(DOTA)]^2−^ and [LaOH(DOTA)]^2−^, where negative charges reduce electrostatics, making orbital interactions dominant. The Δ*E*_int_ values are smaller for [La(H_2_O)L] complexes, implying weaker bonding with water. Higher water stability correlates with greater electrostatic bonding, as seen for [LaF(DOTpy)]^2+^ (78.13% electrostatics) *versus* [La(H_2_O)(DOTpy)]^3+^ (61.40%).

### 3.4. ^18^F-fluorination

Having successfully prepared three [La^19^F(L)]X complexes, we proceeded to attempt their radiofluorination. Considering the varying stabilities of the [La–F] bond in aqueous media, the synthesis of [^18^F][LaF(DOTpy)]OTf_2_ (8) was attempted in organic solution.

Several procedures for radiofluorination of metal complexes have been previously reported, namely direct and indirect radiofluorination.^49^ For our study, we opted for an indirect approach *via* La[^18^F]F intermediate to yield the desired [^18^F][LaF(DOTpy)]OTf_2_ complex. This method was chosen as it has been successfully employed in the synthesis of Al[^18^F]F complexes, demonstrating reliable [^18^F]fluoride incorporation under mild conditions.^50,51^

To enable anhydrous ^18^F-fluorination, the processing of [^18^F]fluoride target water was the first essential step. Anhydrous, no–carrier added [^18^F]fluoride was obtained as [^18^F]BnEt_3_NF in methanol solution using a previously published QMA processing procedure.^27^

**Fig. 6:**
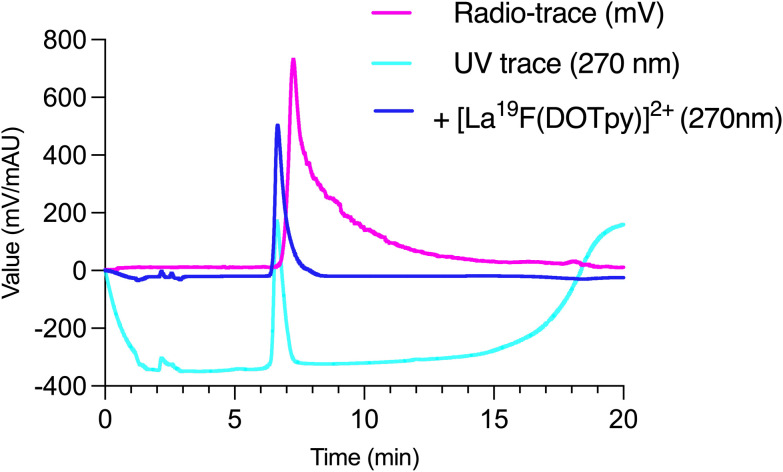
RadioHPLC of [^18^F][LaF(DOTpy)]^2+^ (UV trace – cyan; radio trace – magenta) and [LaF(DOTpy)]^2+^ (UV trace – blue) C-23 RP column H_2_O/MeOH 0–90% 20 min.

A series of radiofluorination reactions *via* pre-formation of the intermediate [^18^F][LaF(OTf)_2_], prepared by adding [^18^F]BnEt_3_NF to a lanthanum triflate precursor was performed. Approximately 1 pmol (100 MBq) of [^18^F]fluoride was reacted with a large excess of La(OTf)_3_ (10 μmol) in methanol to ensure near-complete capture of the radioisotope. Subsequent addition of DOTpy (20 μmol) afforded the [^18^F][LaF(DOTpy)]^2+^ complex under mild conditions. The large metal and ligand excesses were employed to maximize radiochemical yield and suppress competing formation of LaF_3_ precipitate. Radiochemical conversion and purity were assessed by radioHPLC, which indicated complete incorporation of [^18^F]fluoride into the desired [^18^F][LaF(DOTpy)]^2+^ species. The identity of the radiolabeled complex was further confirmed by co-injection of a large excess of non-radioactive reference complex 8, [LaF(DOTpy)]OTf_2_, into the radiolabeling mixture, followed by radioHPLC analysis (Fig. 6).

However, the resulting complex did not demonstrate high stability. When the same sample was re-analyzed by radioHPLC one hour after preparation, a significant amount of free and degraded DOTpy ligand was detected. This observation indicates that, although initial radiochemical incorporation was quantitative, the complex undergoes rapid decomposition under the investigated conditions. These findings underscore the limitations of stabilizing the La–F bond and highlight the need for further ligand optimization to achieve sufficiently robust coordination environments for practical radiofluorination applications.

## 4. Conclusions

In this study, we demonstrated the synthesis of first-in-class La–[^18/19^F]fluoride coordination complexes with four macrocyclic chelators. Among all investigated compounds, fluorinated complexes [La^19^F(L)]^*x*^, where L = macropa or DOTpy, exhibited stability in organic solvent (MeOH) and were successfully isolated and fully characterized. The complex [LaF(DOTpy)]OTf_2_ (8) further showed high stability in aqueous media. DFT/EDA analyses provided mechanistic insight into respective stabilities of [LaF(L)]^*x*^ complexes relative to other scaffolds, with less electronegative ligands resulting in more stable complexes. Systematic evaluation revealed a key trade-off: less electron-donating ligands strengthen La–F bonds but reduce L–La thermodynamic stability. Optimal La–F radiopharmaceuticals will require chelator design that balances these competing stability requirements. Successful radiofluorination was achieved for the [^18^F][LaF(DOTpy)]^2+^ complex *via* a La[^18^F]F intermediate under mild conditions. Stability under formulation conditions, kinetic barriers to hydrolysis, and *in vivo* behavior remain to be established. However, this work serves as a proof-of-concept and opens new possibilities for theranostic approaches beyond La–F chemistry. Specifically, further development and application of this approach to ^225^Ac-radiopharmaceuticals are currently underway, and the future identification of *in vivo* stable ^18^F-labeled La/Ac surrogates could ultimately support dosimetry and therapy planning for ^225^Ac-based targeted alpha therapy, once suitable candidates are available.

## Author contributions

Conceptualization: RF; Synthesis: RF; Radiochemistry: RF; Computation: CL, GS; Data curation: RF; Writing–original draft: RF, CL, GS; Writing–review & editing: RF, MB, SG, LM, CL, GS, MS; Supervision: MB, SG, LM.

## Conflicts of interest

There are no conflicts to declare.

## Supplementary Material

DT-055-D5DT02645H-s001

DT-055-D5DT02645H-s002

## Data Availability

Supplementary information (SI): computational data, including optimized geometries, energy decomposition analysis, and calculated bond distances and reaction energies for the La–F complexes. All experimental procedures, characterization data (^1^H NMR, ^19^F NMR, LC–MS, HR–MS spectra), and ^18^F-radiofluorination experiments. See DOI: https://doi.org/10.1039/d5dt02645h. CCDC 2496547 and 2496407 and 2496543 (5, 6, and 8) contain the supplementary crystallographic data for this paper.^52*a*–*c*^
