# Supplementary material for: La–[18/19F]fluoride complexes: a novel addition to the radiofluorination family
Source: Dalton Trans. 2026 Mar 30;55(15):5944–51. doi: 10.1039/d5dt02645h (PMC13034926; doi:10.1039/d5dt02645h)
Supplement: DT-055-D5DT02645H-s001 [file DT-055-D5DT02645H-s001.pdf]

Supplementary Information (SI) for Dalton Transactions.
This journal is © The Royal Society of Chemistry 2026

Supporting Information

La-[$^{18/19}$F]fluoride complexes: a novel addition to the radiofluorination family.

*Martin Behe, Stefan Gruber, Cen Li, Georg Schreckenbach, Linjing Mu, Margret Schottelius, and Radmila Faizova**

1 Table of Contents

1 Table of Contents 1
2 Materials and Instruments 2
2.1 [La(L)] and [La(L)F] synthesis and characterization 3
^{19}F-Fluorination of [La(NO_3)$_3$(H_2O)$_6$] 3
Synthesis and ^{19}F-fluorination of [La(DOTA)]$^-$ in MeOH 3
Synthesis and ^{19}F-fluorination of [La(HDOTA)] in aqueous buffer 4
Synthesis and ^{19}F-fluorination of [La(macropa)]$^+$ in MeOH 5
Synthesis and ^{19}F-fluorination of [La(macropa)]$^+$ in aqueous buffer. 6
Synthesis and ^{19}F-fluorination of [La(DOTAM)]$^{3+}$ in MeOH. 7
Synthesis and ^{19}F-fluorination of [La(DOTAM)]$^{3+}$ in aqueous buffer. 8
Synthesis and ^{19}F-fluorination of [La(DOTpy)(OTf)]$^{2+}$ in MeOH. 9
Water stability of [LaF(DOTpy)]OTf$_2$ (8) 11
2.2 ^{18}F-fluorination 13
Stability of [LaF(DOTpy)]OTf$_2$ under different conditions 13
^{18}F-fluorination via La[^{18}F]F intermediate 14
2.2 Computational Methods 15
2.3 X-ray crystallography 41
3 References: 42

2 Materials and Instruments

All solvents and reagents, unless otherwise noted, were purchased from commercial sources and used as received without further purification.

Chemicals:

DOTpy (1,4,7,10-tetrakis(2-pyridylmethyl)-1,4,7,10-tetraazacyclododecane) ligand was synthesized according to a previously published procedure.[1]

NMR spectra were recorded at ambient temperature on a 400 MHz (^{1}H) and 376 MHz (^{19}F) spectrometer, chemical shifts are reported in ppm. ^{1}H NMR spectra were referenced to the residual solvent peak. ^{19}F NMR spectra were referenced to a triflate $\delta = -80$ ppm.

Compounds were analyzed by LCMS (Bruker Amazon1 LCMS-system connected to Agilent 1290 infinity II).

For radio-HPLC, a Dionex with a C-23 reversed-phase column (120 BS-C23, 5 µm, 150 × 4.6 mm) and a radiodetector (Berthold, HPLC Radioflow Detector, LB 509); or Agilent 1200 instrument equipped with a Phenomenex Luna C18 reverse phase column (100 × 4.6 mm, 5 µm) and a GABI star radioactive HPLC flow monitor (Elysia-ray test GmbH) was employed.

Gradient HPLC methods were employed using a binary mobile phase that contained H_2O + 0.1 % TFA (A) and MeOH (B): HPLC Method 1: flow rate of 1 mL/min; 0-90% B (0–10 min), 90% B (10–12 min), 90-0% B (14 min), 0% (14–15 min).

For Method 2, gradient HPLC methods with binary mobile phase that contained H_2O (A) and MeOH (B): HPLC Method 2: flow rate of 1 mL/min; 5-30% B (0–10 min), 30-60% B (10–15 min), 60-95% B (15–17 min), 95% (17–18 min), 5% (18–20 min).

[^{18}F]fluoride was obtained by bombardment of 98% enriched (^{18}O) water via the ^{18}O(p,n)^{18}F nuclear reaction in a Cyclone 18/9 cyclotron (18-MeV; IBA). [^{18}F]$BnEt_3NF$ in MeOH was prepared by passing the [^{18}F]fluoride target water through a pre-conditioned QMA Carbonate Plus Light cartridge, followed by a 1 mL wash with the corresponding solvent. [^{18}F]$BnEt_3NF$ was then eluted with 1 mL of a 30 mM $BnEt_3NCl$ solution in MeOH.

2.1 [La(L)] and [La(L)F] synthesis and characterization

^{19}F-Fluorination of $[La(NO_3)_3(H_2O)_6]$

A colourless solution of lanthanum nitrate (10.0 mg, 0.023 mmol, 1 equiv.) in water was treated with a colourless solution of either KF (1.5 mg, 0.025 mmol, 1.1 equiv.), CsF (10.2 mg, 0.025 mmol, 1.1 equiv.), or TBAF (7.9 mg, 0.025 mmol, 1.1 equiv.). Immediate precipitation occurred, indicative of LaF_3 formation. ^{19}F NMR spectra demonstrated disappearance of fluoride from the solution, characteristic of precipitated LaF_3 (Figure S1).

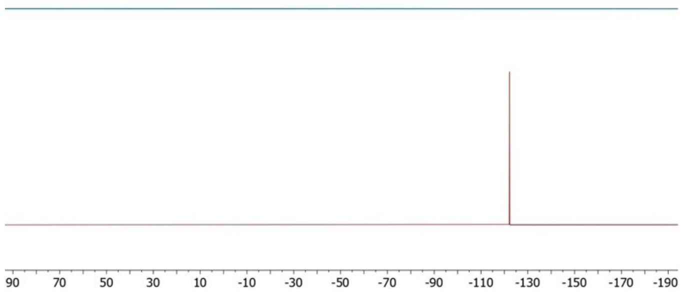

Figure S1. ^{19}F NMR spectrum (376 MHz, D_2O, 298 K) of KF (bottom); the reaction mixture after the addition of KF to $[La(NO_3)_3(H_2O)_6]$ (top).

Synthesis and ^{19}F-fluorination of $[La(DOTA)]^-$ in MeOH.

H_4DOTA (11.4 mg, 0.028 mmol, 1.1 equiv.) was suspended in methanol (0.5 mL), and triethylamine (4.4 equiv.) was added dropwise to yield a colourless solution. A solution of lanthanum triflate (15.0 mg, 0.026 mmol, 1 equiv.) in methanol (0.5 mL) was then added dropwise, and the mixture was stirred at 60 °C for 12 hours. After cooling to room temperature, tetrabutylammonium fluoride (TBAF, 8.2 mg, 0.026 mmol, 1 equiv.) was added, and the reaction mixture was analyzed by ^{19}F NMR, which showed a resonance at δ = –144.8 ppm attributed to free fluoride. After stirring for 30 minutes, a second equivalent of TBAF (8.2 mg, 0.026 mmol, 1 equiv.) was added, and the mixture was stirred for an additional 30 minutes. ^{19}F NMR and mass spectrometry analysis confirmed the absence of fluorinated species.

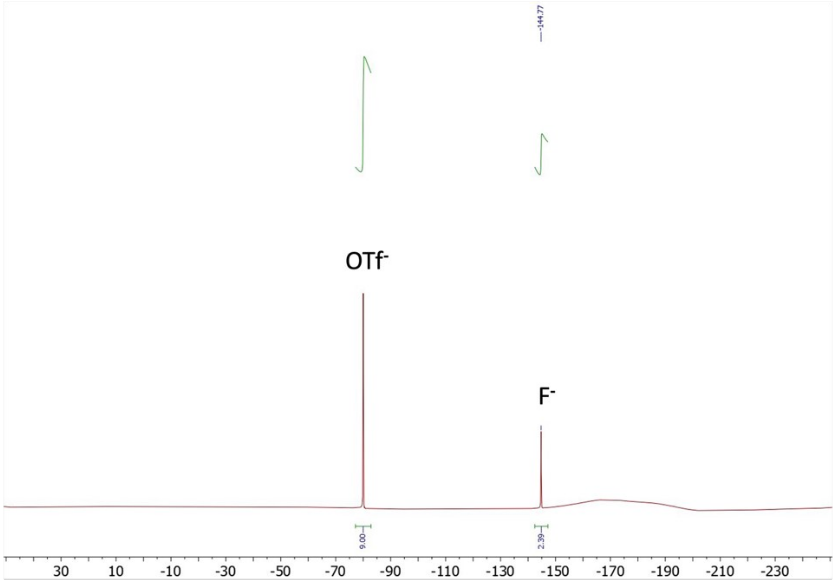

Figure S2. ^{19}F NMR spectrum (376 MHz, MeOD/MeOH, 298 K) after the addition of 2 equiv. of TBAF to $[La(DOTA)]^{-}$.

Synthesis and ^{19}F-fluorination of [La(HDOTA)] in aqueous buffer.

A colourless solution of lanthanum triflate (32.3 mg, 0.055 mmol, 1 equiv.) in NH_4OAc (pH=5; 2.0 mL) was added dropwise to a colourless solution of H_4DOTA (22.2 mg, 0.055 mmol, 1 equiv.) in NH_4OAc (pH=5; 1.0 mL). The resulting solution was stirred for 20 hours at 80 °C.
TOF MS: Found *m/z* 540.3, 558.08; Calculated for $[C_{16}H_{25}N_4O_8La]^+$ ($[La(HDOTA)]^+$) and $[C_{16}H_{27}N_4O_9La]^+$ ($[La(H_2O)(HDOTA)]^+$), respectively: 540.3, 558.08.
To the reaction mixture a colourless solution of TBAF (120 mg, 0.38 mmol, 7 equiv.) in NH_4OAc (1.4 mL) was prepared and stepwise added to the solution of the complex at room temperature. The ^{19}F NMR indicated only a resonance of free fluoride (δ= −122 ppm), even after the addition of 7 equiv. of TBAF (Figure S3). Mass spectrometry analysis of the reaction mixture revealed no detectable $[LaF(HDOTA)]^{2-}$ ion.

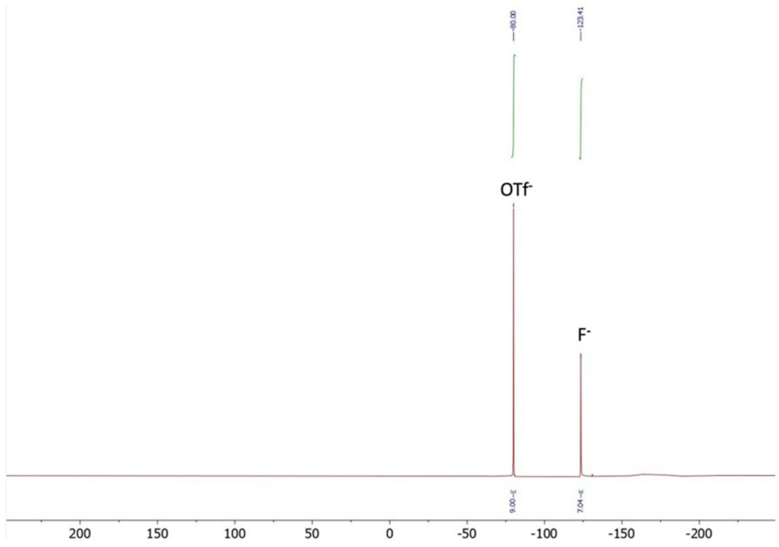

Figure S3. ^{19}F NMR spectrum (376 MHz, D_2O/H_2O, 298 K) after the addition of 7 equiv. of TBAF to [La(HDOTA)].

Synthesis and ^{19}F-fluorination of $[La(macropa)]^+$ in MeOH.

To a colourless suspension of H_2macropa (41.2 mg, 0.08 mmol, 1.1 equiv.) in methanol (1 mL), triethylamine (TEA, 2.2 equiv.) was added dropwise, resulting in a colourless solution. A colourless solution of lanthanum triflate (42.8 mg, 0.07 mmol, 1 equiv.) in methanol (0.5 mL) was added dropwise to the ligand solution. The resulting mixture was stirred for 1 hour at ambient temperature. LC-MS (*m/z*): Found 669.2; Calculated for $[C26H34N4O8La]^+$ 669.14.

A colourless solution of TBAF (22.1 mg, 0.07 mmol, 1 equiv.) was added to the reaction mixture, and a ^{19}F NMR spectrum was recorded. The spectrum showed a resonance corresponding to triflate (δ = –80 ppm), and a new resonance at δ = –7 ppm, assigned to La-bound fluoride.

Addition of further equivalents of TBAF led to the appearance of a free fluoride resonance in the ^{19}F NMR spectrum (Figure 1, Figure S4).

Stability of the complex in methanol was monitored by ^{19}F NMR over two weeks, revealing increasing formation of insoluble LaF_3. The stability of [LaF(macropa)] in water was also evaluated: upon addition of 300 µL (50% v/v) HEPES buffer (pH=7), the resonance assigned to the La–F bond completely disappeared (Figure S5).

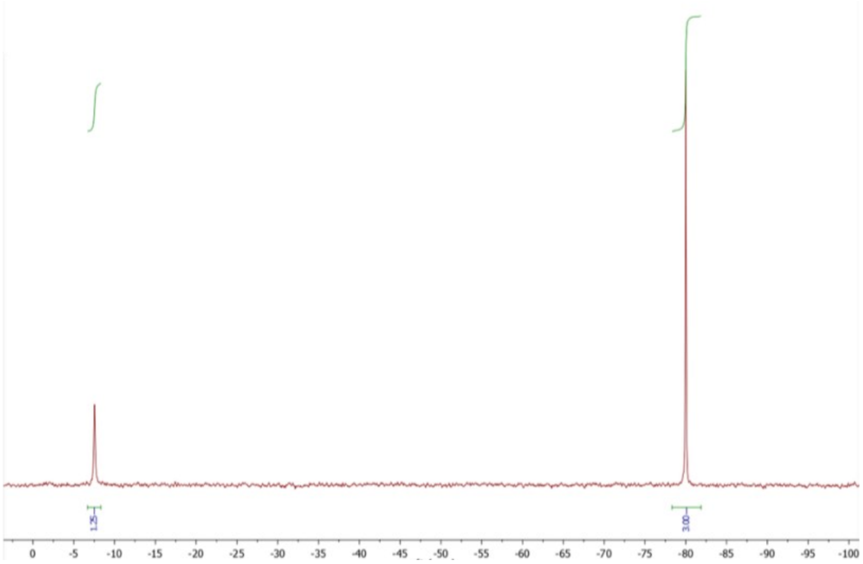

Figure S4. ^{19}F NMR spectra (376 MHz, MeOD, 298 K) of [LaF(macropa)] recorded immediately after the addition of 1 equiv. of TBAF to [La(macropa)]OTf in MeOD.

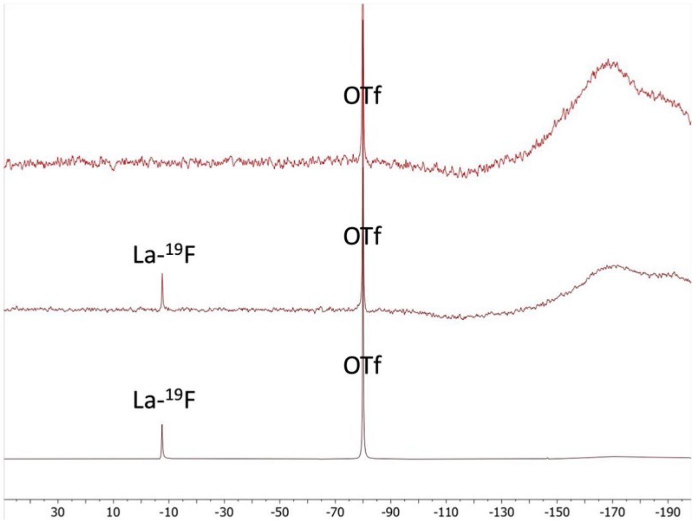

Figure S5. ^{19}F NMR spectra (376 MHz, MeOH or MeOH/H_2O, 298 K) of *in situ* prepared [LaF(macropa)] recorded immediately after the synthesis (bottom); 2 weeks after the synthesis (middle); and after the addition of 300 μL (50% by volume) of aq. HEPES (pH=7)**.**

Synthesis and ^{19}F-fluorination of $[La(macropa)]^+$ in aqueous buffer.

A colourless solution of lanthanum triflate (12.2 mg, 0.02 mmol, 1 equiv.) in 0.25 M NH_4OAc (pH = 5; 1.0 mL) was added dropwise to a colourless solution of H_2macropa (11 mg, 0.02 mmol, 1.1 equiv.) in 0.25 M aqueous NH_4OAc (pH = 5; 0.5 mL). The resulting mixture was stirred for 1 hour at 60 °C. Subsequently, a colourless solution of TBAF (5.9 mg, 0.02 mmol, 1 equiv.) was added to the reaction mixture at room temperature and stirred overnight. The ^{19}F NMR spectrum showed

resonances corresponding to free fluoride and triflate. Upon addition of a second equivalent of TBAF (5.9 mg, 0.02 mmol, 1 equiv.), a further ^{19}F NMR spectrum was recorded, again only indicated free fluoride and triflate resonances.

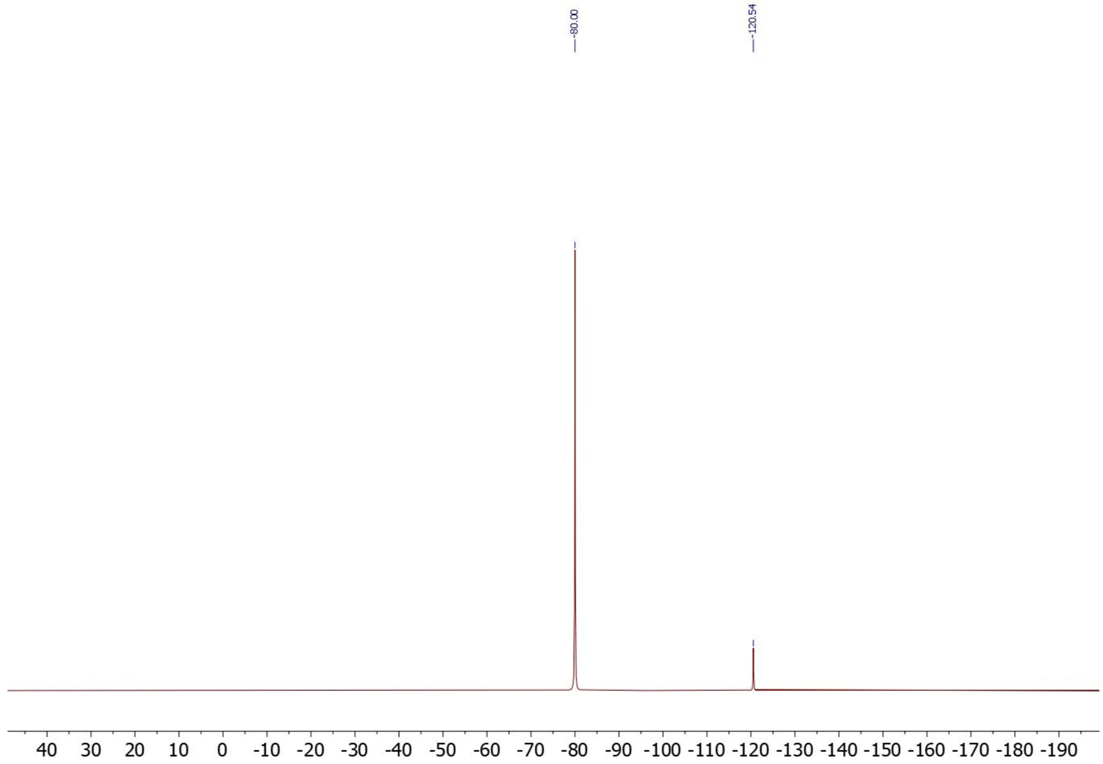

Figure S6. ^{19}F NMR spectrum (376 MHz, D_2O, 298 K) after the addition of 2 equiv. of TBAF to [La(macropa)]OTf.

Synthesis and ^{19}F-fluorination of $[La(DOTAM)]^{3+}$ in MeOH.

To a colourless suspension of DOTAM (7.0 mg, 0.017 mmol, 1.1 equiv.) in MeOH (0.5 mL), a colourless solution of lanthanum triflate (9.4 mg, 0.016 mmol, 1 equiv.) in MeOH (0.5 mL) was added dropwise to the solution of the ligand. The resulting solution was stirred for 12 hours at 60 °C. LC-MS (*m/z*): Found 270.65; Calculated for $[C16H32N8O4La]^{2+}$ 270.02

To the reaction mixture, a colourless solution of TBAF (5.4 mg, 0.017 mmol, 1.1 equiv.) in MeOH (0.5 mL) was added and a reaction mixture was stirred at room temperature for 2 hours. LC-MS (*m/z*): Found 280.2; 559.6 Calculated for $[C16H32N8O4LaF]^{2+}$ 279.08; 558.16. ^{19}F NMR spectrum was recorded, showing a resonance of triflate (δ= −80 ppm), a new resonance assigned to a La–F (δ= −13.7 ppm)(Figure S7). Further, precipitation was observed, suggesting the formation of LaF_3.

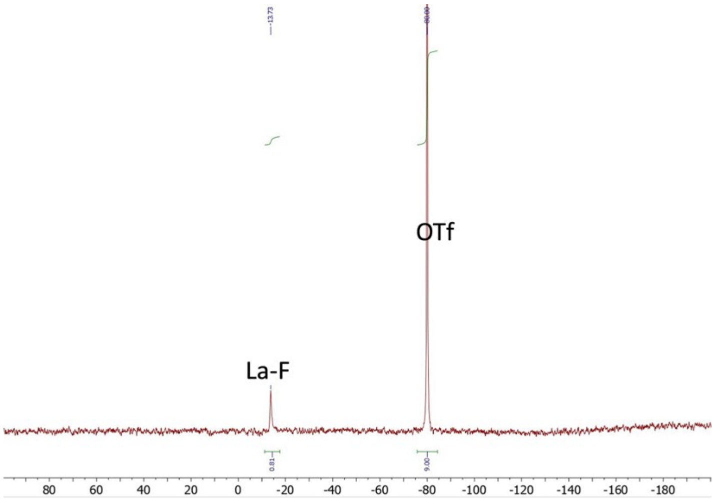

Figure S7. ^{19}F NMR spectrum (376 MHz, MeOD, 298 K) after the addition of TBAF to $[La(DOTAM)]^{3+}$.

Synthesis and ^{19}F-fluorination of $[La(DOTAM)]^{3+}$ in aqueous buffer.

A colourless solution of lanthanum triflate (21.2 mg, 0.036 mmol, 1 equiv.) in 0.25 M NH_4OAc (pH=5; 0.5 mL) was added dropwise to a colourless solution of DOTAM (14.5 mg, 0.036 mmol, 1 equiv.) 0.25 M NH_4OAc (pH=5; 0.5 mL). The resulting solution was stirred for 12 hours at 80 °C. LC-MS (*m/z*): Found 270.65; Calculated for $[C16H32N8O4La]^{2+}$ 270.02.

To the reaction mixture at ambient temperature, a colourless solution of TBAF (11.4 mg, 0.036 mmol, 1 equiv.) in 0.25 M NH_4OAc (pH=5; 0.5 mL) was added and left stirring for 1 hour at room temperature. Upon the addition of the TBAF solution, the formation of precipitate was observed.

A ^{19}F NMR spectrum was recorded which indicated a broad resonance (δ= −160 ppm) and a new resonance (δ= −20.5 ppm) that was assigned to a "La–F" bond (Figure S8).

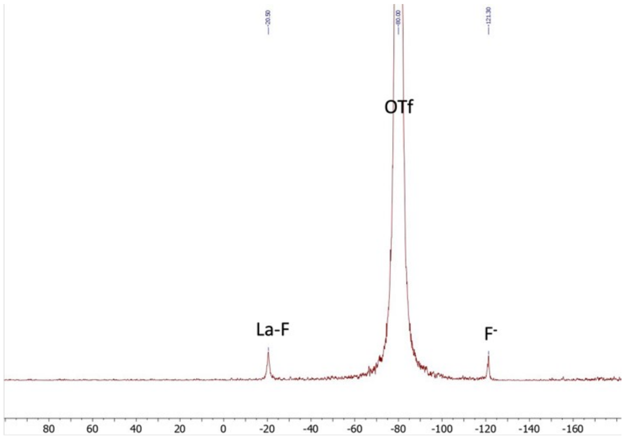

Figure S8. ^{19}F NMR spectrum (376 MHz, H_2O, 298 K) after the addition of 1 equiv. of TBAF to complex **3** in aq. NH_4OAc (pH=5).

Synthesis and ^{19}F-fluorination of $[La(DOTpy)(OTf)]^{2+}$ in MeOH.

^{19}F NMR indicated a resonance of triflate (δ= −80 ppm) and a new resonance (δ= 34 ppm) that was assigned to a "La–F" bond (Figure S11). The reaction mixture was crystallized by Et_2O vapour diffusion, resulting in colourless crystals (130.0 mg; 74%) of $[LaF(DOTpy)][OTf]_2$ (**8**) suitable for Xray diffraction Anal. Calcd for $C_{34}H_{40}N_8F_7LaO_6S_2$ (**8**): C, 41.12; H 4.06; N, 11.29. Found: C, 41.41; H 4.41; N, 11.16.

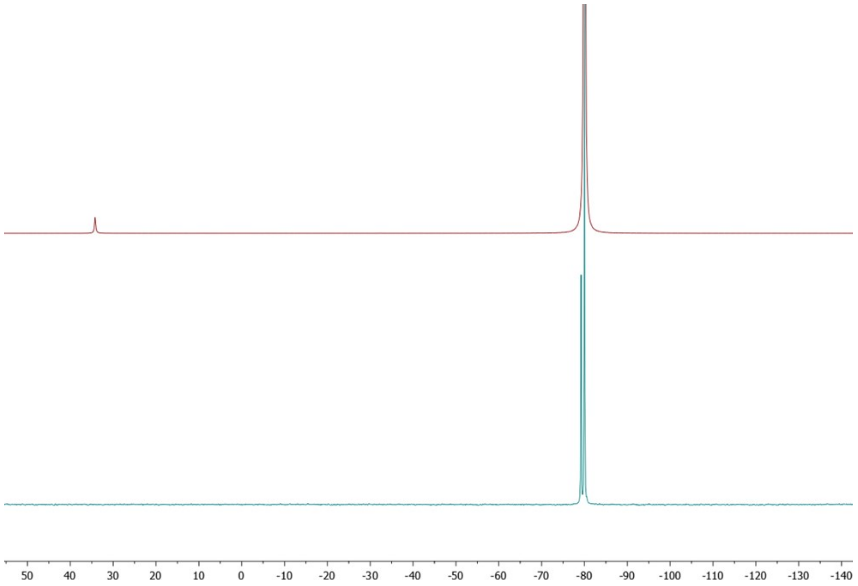

Figure S9. ^{19}F NMR spectrum (376 MHz, MeOD, 298 K) of $[La(DOTpy)(OTf)]OTf_2$ **4** (bottom) and the reaction mixture after the addition of TBAF to 4 (top).

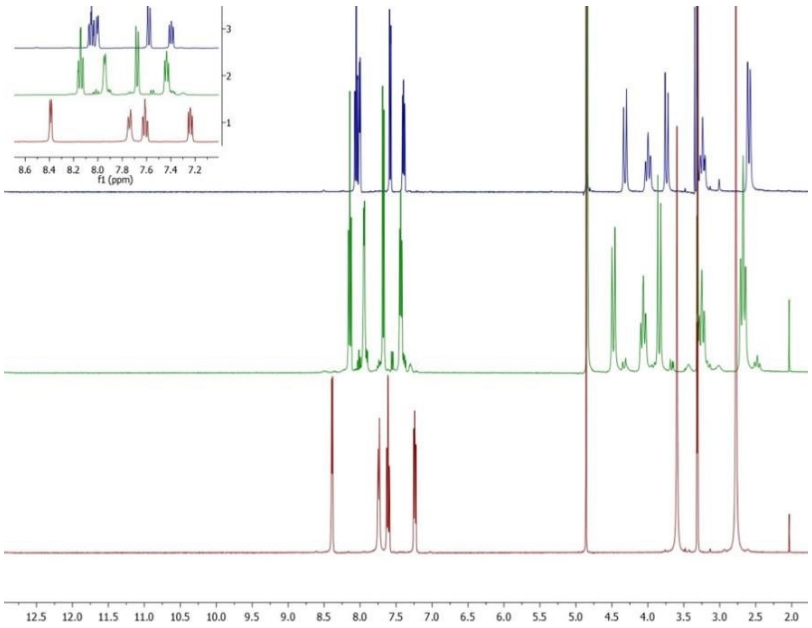

Figure S10. ^1H NMR spectra (400 MHz, MeOD, 298 K) of DOTpy ligand (bottom); $[La(DOTpy)(OTf)]OTf_2$ (**4**) (middle); and $[LaF(DOTpy)]OTf_2$ (**8**) (top).

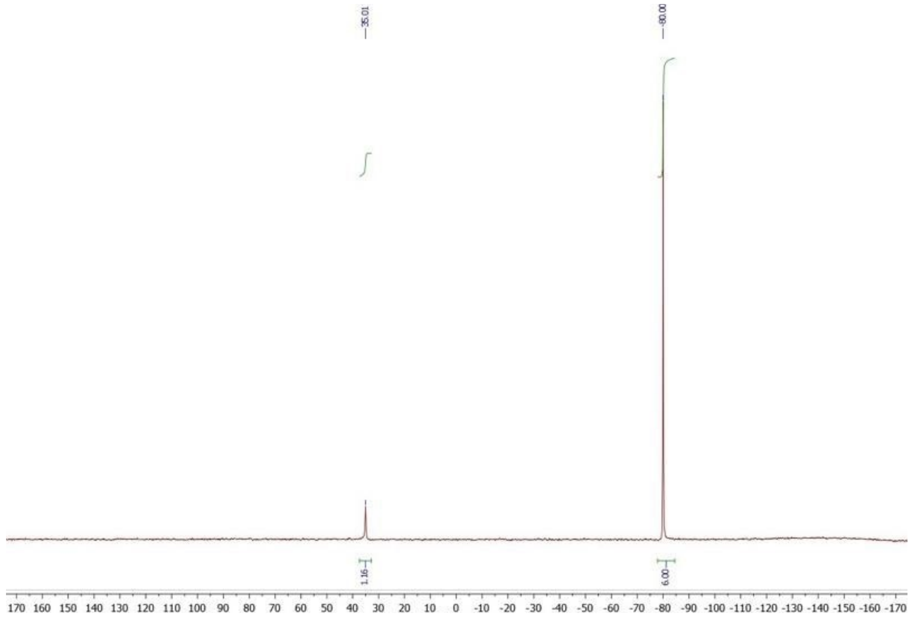

Figure S11. ^{19}F NMR spectrum (376 MHz, MeOD, 298 K) of crystals of [LaF(DOTpy)]OTf_2 **(8).**

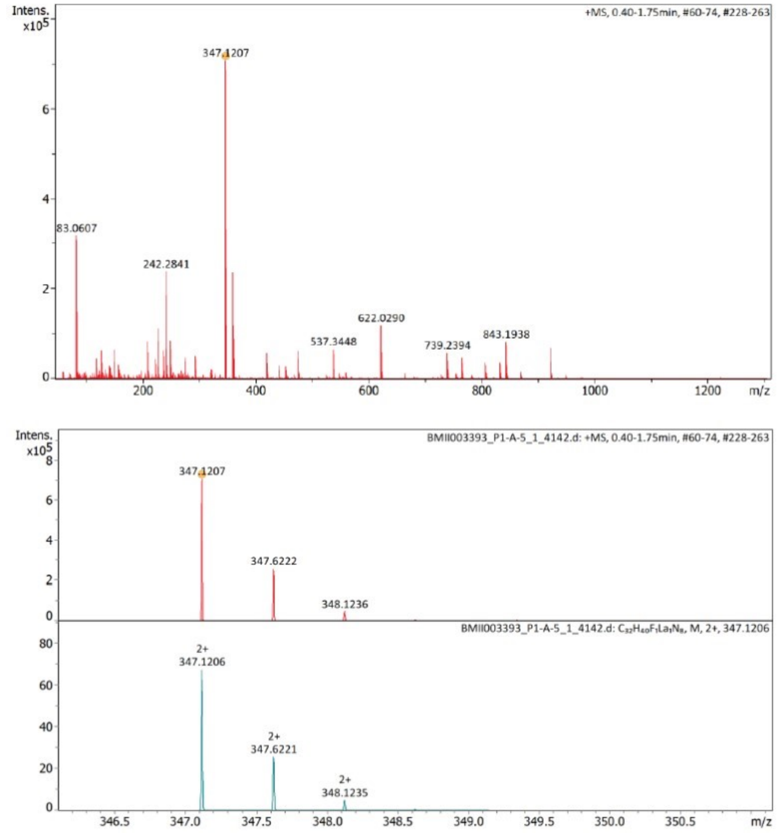

Figure S12. HR-MS: (C32H40FLaN8) $^{2+}$ *m/z*= 347.1206 $^{2+}$ found: 347.1207

Water stability of [LaF(DOTpy)]OTf$_2$ (8).

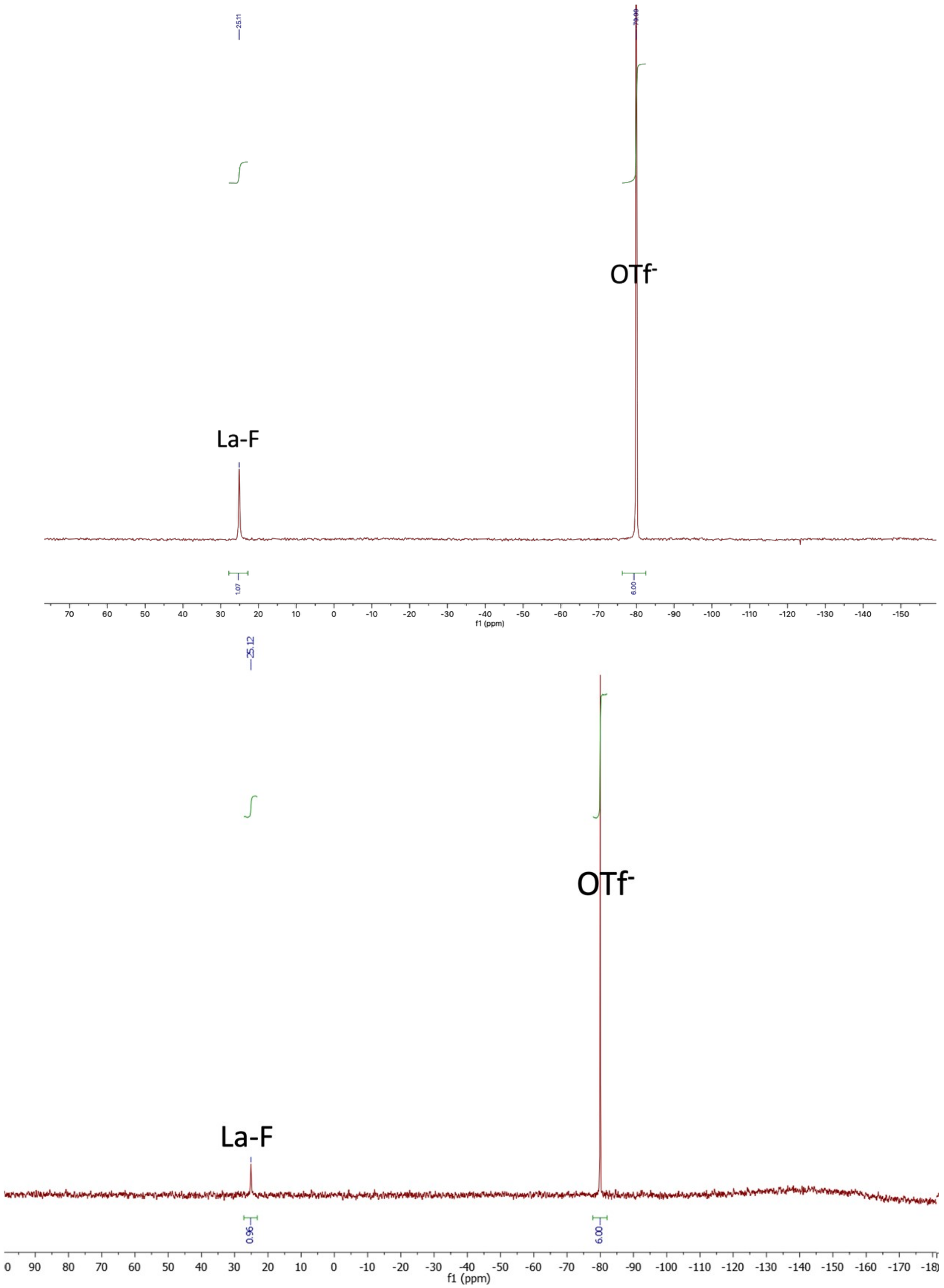

Figure S13. ^{19}F NMR spectra (376 MHz, D_2O, pH7, 298 K) of [LaF(DOTpy)]OTf$_2$ immediately (top) and 3 weeks (bottom) after the addition of D_2O.

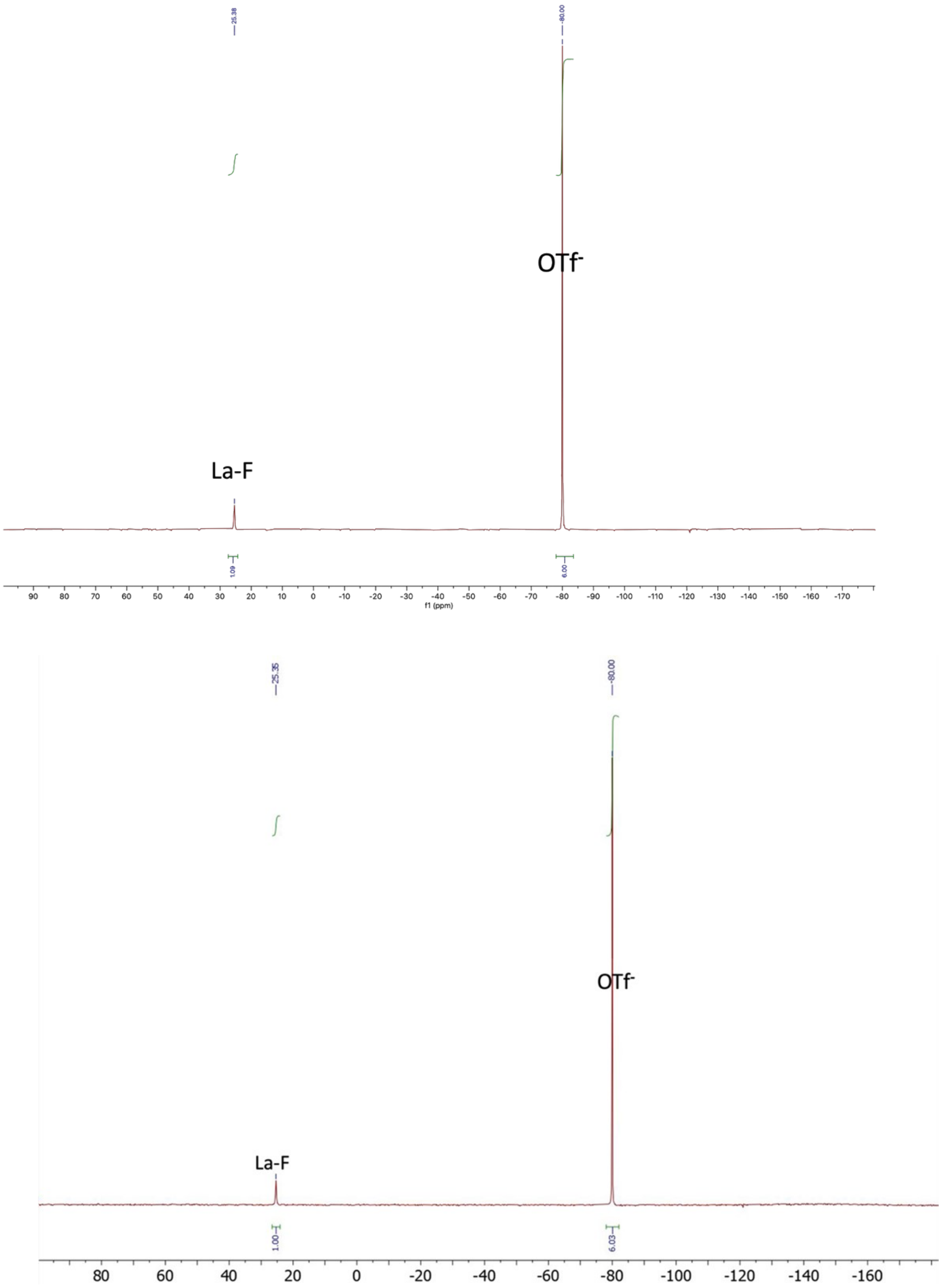

Figure S14. ^{19}F NMR spectra (376 MHz, D_2O, pH5, 298 K) of [LaF(DOTpy)]OTf_2 immediately (top) and 3 weeks (bottom) after the addition of D_2O.

2.2 ^{18}F-fluorination

Stability of [LaF(DOTpy)]OTf$_2$ under different conditions.

Firstly, the stability of complex **8** was evaluated using HPLC. A methanol solution of complex **8** was analyzed with Method 1 immediately after dissolution and repeated 1 day later. HPLC chromatograms monitored at 270 nm indicated that the complex **8** remains stable in MeOH for at least 1 day (Figure S15). Further, the solution of **8** was passed through an N-alumina cartridge and analyzed by HPLC, demonstrating the stability of the complex following N-alumina purification (Figure S16). Lastly, an HPLC fraction was collected between 5.4 and 6 minutes (pH=4) and was reinjected into the HPLC using Method 1. The HPLC chromatogram suggested instability of [LaF(DOTpy)]OTf$_2$ in the HPLC eluent (Figure S17). For the following radiofluorination experiments no TFA was added to the HPLC eluents.

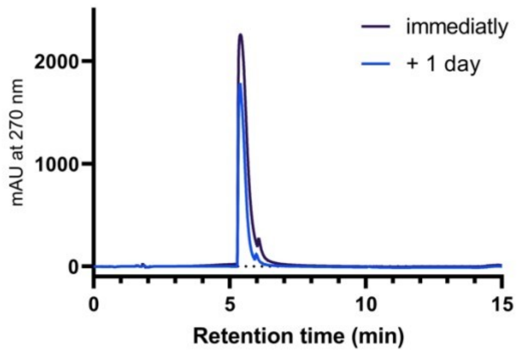

Figure S15. HPLC chromatograms of [LaF(DOTpy)]OTf$_2$ with Method 1 immediately and 1 day after the dissolution in MeOH.

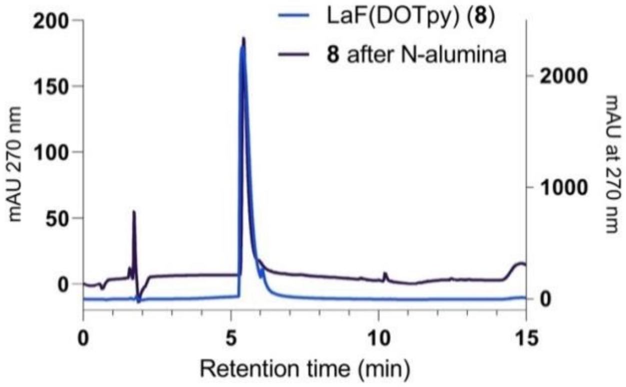

Figure S16. HPLC chromatograms of [LaF(DOTpy)]OTf$_2$ (8; blue) and 8 after N-alumina cartridge purification (purple) with Method 1**.**

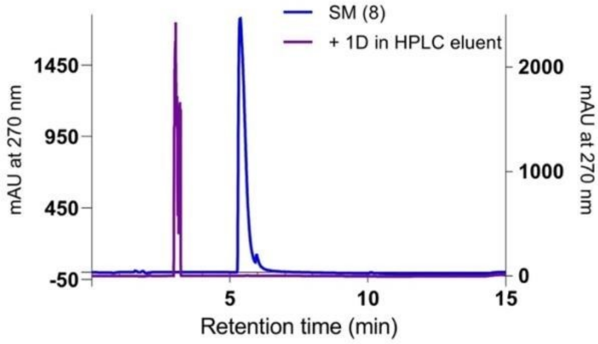

Figure S17. HPLC chromatograms of [LaF(DOTpy)]OTf_2 (blue) and reinjected HPLC fraction (5.4–6 minutes; purple) with Method 1.

^{18}F-fluorination via La[^{18}F]F intermediate.

To 100 µL of a 10 mM stock solution of $La(OTf)_3(H_2O)_2$ in MeOH 100 µL of [^{18}F]$BnEt_3NF$ in methanol (50-100 MBq) were added. The reaction mixture was left at 60 °C for 30 minutes, resulting in [^{18}F][LaF$(OTf)_2$]. Then, 20 µL of a 100 mM stock solution of DOTpy ligand in methanol were added to the reaction mixture. The reaction mixture was left at 60 °C for 5 minutes resulting in [^{18}F][LaF(DOTpy)] (MA = 0.5-0.2 GBq/µmol 35 mins post-EOS). The product was analyzed by radioHPLC (Figure S18).

The identity of the radiolabeled complex was further confirmed by co-injection of a large excess of non-radioactive reference complex **8**, [LaF(DOTpy)]OTf_2, into the radiolabeling mixture, followed by radioHPLC analysis.

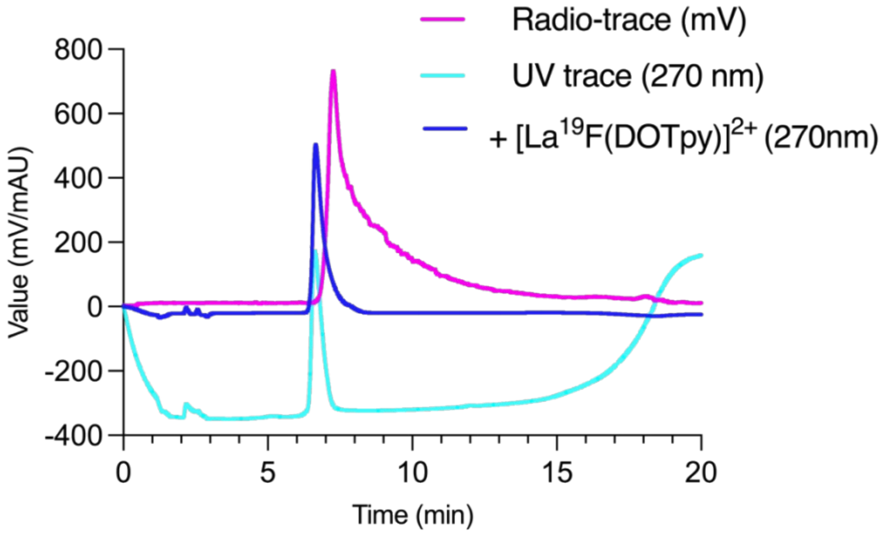

Figure S18. Radio-HPLC chromatogram of [^{18}F][LaF(DOTpy)]$^{2+}$ by indirect radiofluorination approach.

2.2 Computational Methods

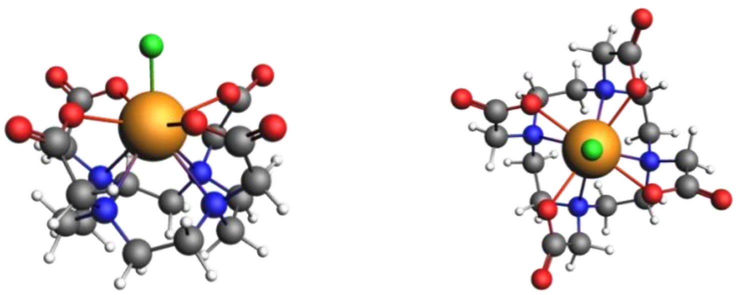

(a)

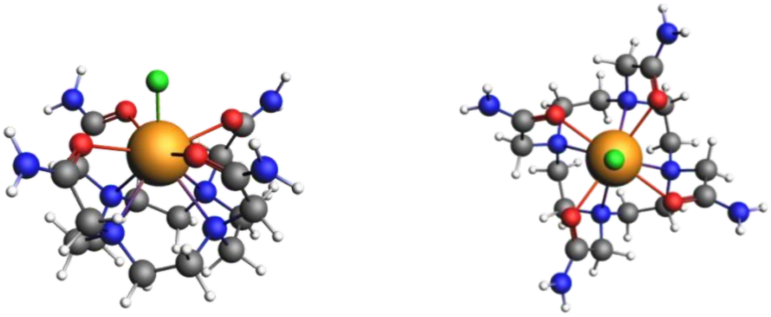

(b)

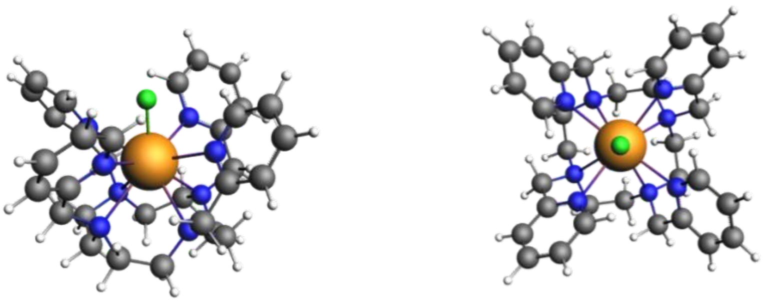

(c)

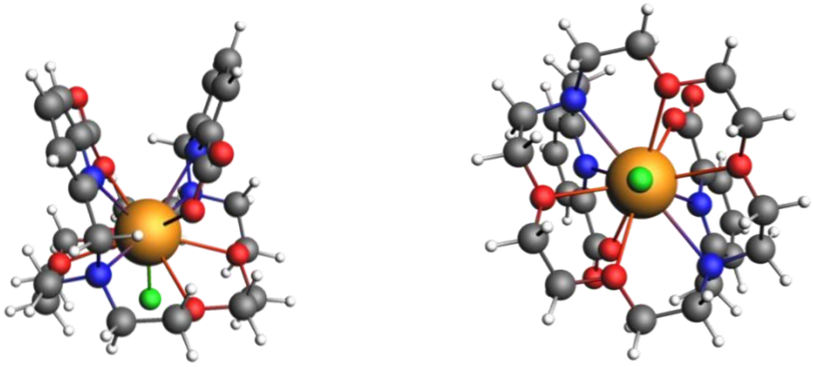

(d)

Figure S19. Optimized geometries of (a) $[LaF(DOTA)]^{2-}$ (b) $[LaF(DOTAM)]^{2+}$ (c) $[LaF(DOTpy)]^{2+}$ (d) $[LaF(macropa)]^{0}$ at PBE-D3(BJ)/TZ2P level of theory under aqueous condition. (Left: side view; Right: top view.) (Yellow: Lanthanum; Grey: carbon; White: Hydrogen; Blue: Nitrogen; Red: Oxygen; Green: Fluorine).

Complex	Bond type	Distance
**La-X-DOTA**	La-F	2.281
	La-O (OH^-)	2.335
	La-O (H_2O)	2.759
**La-X-DOTAM**	La-F	2.227
	La-O (OH^-)	2.268
	La-O (H_2O)	2.632
**La-X-DOTpy**	La-F	2.216
	La-O (OH^-)	2.252
	La-O (H_2O)	2.644
**La-X-Macropa**	La-F	2.239
	La-O (OH^-)	2.304
	La-O (H_2O)	2.649

Table S1. La-X distances (Å) in the optimized structures of [LaX(L)] (X=F^-, OH^- and H_2O; L=$DOTA^{4-}$, DOTAM, DOTpy and $macropa^{2-}$)

	La complexes	Formation Reaction Energy	Ligand Substitution Reaction Energy
**DOTA**	$LaDOTA^-$	-105.72	7.00
	$La(H_2O)DOTA^-$	-105.94	6.78
	$La(OH)DOTA^{2-}$	-113.34	-0.62
	$LaFDOTA^{2-}$	-112.72	0
**DOTAM**	$LaDOTAM^{3+}$	-65.95	17.82
	$La(H_2O)DOTAM^{3+}$	-67.91	15.86
	$La(OH)DOTAM^{2+}$	-86.97	-3.20
	$LaFDOTAM^{2+}$	-83.77	0
**DOTpy**	$LaDOTpy^{3+}$	-65.95	25.54
	$La(H_2O)DOTpy^{3+}$	-69.83	21.66
	$La(OH)DOTpy^{2+}$	-92.74	-1.25
	$LaFDOTpy^{2+}$	-91.49	0
**Macropa**	$LaMacropa^+$	-90.01	7.14
	$La(H_2O)Macropa^+$	-89.10	8.05
	La(OH)Macropa	-99.83	-2.68
	LaFMacropa	-97.15	0

Table S2. Calculated ΔG and ΔΔG (in kcal/mol) of complex formation reactions (R1) and axial ligand substitution reactions (R2) relative to [LaFL] complexes.

**Fragments**		**Energy terms**							
		**ΔE_{int}**	**ΔE_{Pauli}**	**ΔE_{elstat}**		**ΔE_{orb}**		**ΔE_{disp}**	
**[LaDOTA]$^-$**	**F^-**	-4.64	73.20	-9.74	(12.51%)	-67.06	(86.15%)	-1.03	(1.32%)
	**OH^-**	-8.52	92.11	-26.55	(26.38%)	-72.26	(71.81%)	-1.81	(1.80%)
	**H_2O**	-16.56	33.71	-32.48	(64.61%)	-15.20	(30.24%)	-2.59	(5.15%)
**[LaDOTAM]$^{3+}$**	**F^-**	-271.31	89.04	-279.10	(77.45%)	-80.15	(22.24%)	-1.09	(0.30%)
	**OH^-**	-277.23	111.77	-297.02	(76.35%)	-90.10	(23.16%)	-1.88	(0.48%)
	**H_2O**	-20.31	25.44	-28.66	(62.64%)	-14.72	(32.17%)	-2.37	(5.18%)
**[LaDOTPy]$^{3+}$**	**F^-**	-283.27	100.87	-300.12	(78.13%)	-82.05	(21.36%)	-1.97	(0.51%)
	**OH^-**	-287.27	121.54	-315.10	(77.08%)	-90.35	(22.10%)	-3.37	(0.82%)
	**H_2O**	-18.36	29.49	-29.38	(61.40%)	-14.27	(29.82%)	-4.21	(8.80%)
**[LaMacropa]$^+$**	**F^-**	-157.01	94.08	-176.00	(70.09%)	-74.07	(29.50%)	-1.92	(0.76%)
	**OH^-**	-161.15	122.13	-198.59	(70.10%)	-81.20	(28.66%)	-3.49	(1.23%)
	**H_2O**	-15.87	36.14	-33.07	(63.58%)	-14.65	(28.17%)	-4.28	(8.23%)

Table S3. EDA results (in kcal/mol) between fragments LaL (L=DOTA, DOTAM, DOTPy and Macropa) and fragments X (X = F^-, OH^- and H_2O) for [LaXL] complexes under gas phase using structures optimized in aqueous phase. * Values in percentage show the contribution of each attractive energy term to the total attractive energy term (ΔE_{elstat} +ΔE_{orb} +ΔE_{disp}).

Coordinates for La complexes

LaDOTA$^-$

La	0.02795578580088	-0.06364508057024	-0.08826244445213
N	2.08678402368219	-0.47560157613946	-1.86421902705838
C	2.14072930720928	0.57143229370796	-2.91114238697827
H	1.41967751464677	0.30575015553750	-3.69352508232694
H	3.13266237848390	0.58628371841500	-3.39658984083134
C	1.95774162601462	-1.82157605663209	-2.47674929455814
H	2.24928463699601	-2.55815487537739	-1.71778853011899
H	2.66888571195408	-1.93259699364225	-3.31434607721929
C	3.30161269287804	-0.44711675507568	-1.02741515824694
C	3.14421438695124	-1.22625441764300	0.28846778998166
O	1.93959108372834	-1.39431017445777	0.71703115777250
O	4.17707375094541	-1.60987641295466	0.87266412846270
H	3.51954260942883	0.59205191982520	-0.74324353347852
H	4.17957509441321	-0.82480181297594	-1.57868535787438
N	0.47769154235867	2.06356084278690	-1.77620417168500

C	-0.55036101937524	2.15949663660498	-2.83872965493765
H	-0.27052998695716	1.47000558407864	-3.64427478022053
H	-0.55679580254703	3.16994359873026	-3.28444708783556
C	1.83478026691045	1.95638191878831	-2.36834095829637
H	2.55740681362531	2.21783229010963	-1.58541952384618
H	1.96167321233830	2.69876647793118	-3.17603258412110
C	0.43418133606091	3.24477258214697	-0.89330426145349
C	1.18896129164492	3.03454716940883	0.42926271004458
O	1.34490998956599	1.81386224253699	0.81485363393116
O	1.56598281437073	4.04289069619690	1.05880662060415
H	-0.61003411477689	3.45241656340683	-0.62002145335153
H	0.82228475142088	4.14321386040439	-1.40279433208531
N	-2.06236565815964	0.45369053690582	-1.79687596481604
C	-2.13673468476572	-0.53142781713199	-2.90089775090852
H	-1.43075583097891	-0.22098988926566	-3.68053096144384
H	-3.13788159982526	-0.51909594093419	-3.36711325972119
C	-1.94473821874370	1.83302704634164	-2.33299152293814
H	-2.22019037552101	2.52435896981079	-1.52697369011514
H	-2.67252459927910	1.99220583195475	-3.14823596559741
C	-3.26103271879270	0.37465314971895	-0.94024502952878
C	-3.07880179909840	1.07526820728369	0.41585568840262
O	-1.86620989803174	1.21758605917999	0.83093194742182
O	-4.10020349361943	1.42546349433892	1.03981511772128
H	-3.47288914263141	-0.67973721515130	-0.71304834578541
H	-4.14952963884757	0.78252625559417	-1.45173582234093
N	-0.45304015901164	-2.08520931988945	-1.88510789327152
C	0.55327852292818	-2.11922100347655	-2.97197272777780
H	0.25775066518111	-1.38491804905618	-3.73101603459642
H	0.55011971151014	-3.10250078283377	-3.47493077483587
C	-1.82110572839506	-1.94507443289437	-2.44415445252039
H	-2.52899899318601	-2.25198604870233	-1.66429269631909
H	-1.96338085121316	-2.63961802522424	-3.29089201723100
C	-0.39268395544564	-3.31643256836290	-1.07423539121413
C	-1.12023134355075	-3.18621969324753	0.27370002871859
O	-1.27064799149460	-1.99049239602383	0.73239939882577
O	-1.48277805603792	-4.23065909734793	0.85080318453709
H	0.65667966725216	-3.53915093109188	-0.83455674419087
H	-0.79119552801485	-4.18356073564259	-1.62799882029541

La(H_2O)DOTA$^-$

La	-0.03765350856711	-0.10899786068056	-0.08097660323210
N	2.06147669452247	-0.50357177045944	-1.83453487644359
C	2.14023127252407	0.56256797598009	-2.85998278824109
H	1.43562809771269	0.31391856749815	-3.66249267805326
H	3.14218628678912	0.58272908547393	-3.32436543969828
C	1.93584312776500	-1.83602003452763	-2.47638931351616
H	2.21034156653511	-2.59004254466363	-1.72856567506526
H	2.65979143774295	-1.93402567936357	-3.30486355785608

C	3.26608646426244	-0.49746313449189	-0.98200591758531
C	3.09720581908972	-1.32380103191919	0.30244184505671
O	1.88781372490035	-1.51117736987881	0.70842316569525
O	4.12425594612550	-1.72465251793267	0.88492614574710
H	3.47380780226811	0.53286455531547	-0.66058551700443
H	4.15267802471321	-0.85176214763806	-1.53548850147422
N	0.45815630345350	2.04518010396932	-1.73896496926386
C	-0.54174922040100	2.14895553474439	-2.82678913338418
H	-0.24514774394171	1.46005857152428	-3.62671589284368
H	-0.53134707954057	3.16045449238502	-3.27060383304599
C	1.82833793388701	1.93942728357534	-2.29903290788922
H	2.53288771419930	2.18066908017036	-1.49368567503382
H	1.97999590487999	2.69553896475078	-3.09006072542018
C	0.39439992552808	3.22566619924553	-0.85638666625113
C	1.12462728171615	3.02464800064584	0.48004631593420
O	1.31504149441352	1.80280626334126	0.85034825439340
O	1.45187090468142	4.03468046211011	1.13141068034800
H	-0.65520957879483	3.42943298146485	-0.60253188813393
H	0.78904881196235	4.12572291875173	-1.35863127534257
N	-2.08944501410651	0.44311275010657	-1.84239900691854
C	-2.14974902727484	-0.52878748464875	-2.95835815087801
H	-1.43195605932487	-0.21126145031590	-3.72418469166978
H	-3.14384111904518	-0.50896436924595	-3.43954053252272
C	-1.95101270854918	1.82661836673592	-2.36095938046575
H	-2.24316155502350	2.51232689502426	-1.55627594477450
H	-2.65586229882922	1.99848400362289	-3.19384093146205
C	-3.30443619177127	0.36521303579802	-1.00903182816708
C	-3.14254826328058	1.07351896739045	0.34604441038182
O	-1.93736300005508	1.21286484457654	0.78090821213669
O	-4.17332352685307	1.43324249983773	0.94981039949165
H	-3.52002365784391	-0.68876233955197	-0.78334653794100
H	-4.18326978717454	0.77302571798122	-1.53734073546861
N	-0.48726703482596	-2.10061714779011	-1.93325715625060
C	0.53817168306399	-2.11565891556442	-3.00145026613636
H	0.26033390886175	-1.36263724551223	-3.74879232486217
H	0.53955578303089	-3.08710680040711	-3.52732948671930
C	-1.84478590852546	-1.94910687877804	-2.51377894486738
H	-2.56684622050786	-2.26177208175697	-1.74944703304119
H	-1.97516623107807	-2.63324884453251	-3.37123426519132
C	-0.44425625314261	-3.34545383176093	-1.14244592958866
C	-1.20975854536766	-3.23425509196095	0.18604362357064
O	-1.37282255378967	-2.04573045423086	0.65718106987324
O	-1.58892001122898	-4.28684438911423	0.73788185505198
H	0.59890800822329	-3.56969157909099	-0.87914668263989
H	-0.82798321579952	-4.20386922065150	-1.71997176453069
O	0.45935824370592	-0.05133220215489	2.63194353975875
H	0.92739003560023	0.79636936267742	2.44693219001744
H	1.16587511248465	-0.72688306607372	2.55869372141707

La(OH)DOTA^{2-}

La	0.01935885428462	-0.02488421226851	0.12543472507792
N	2.08735626417825	-0.47036553138372	-1.81600916074041
C	2.14033847289695	0.56447076635071	-2.86825197861303
H	1.42173799311433	0.29045629597489	-3.64992705405530
H	3.13348385625160	0.57752054389879	-3.35555186616733
C	1.94752533954590	-1.81241245953788	-2.42505577263938
H	2.23671165108677	-2.55291040198870	-1.66979786185002
H	2.65145579074392	-1.93031058284216	-3.27026852083216
C	3.30674385937336	-0.44502892173964	-0.99372114413497
C	3.17889470230635	-1.30850514263269	0.27219273761221
O	1.99254992483838	-1.53379096367192	0.69736650341805
O	4.23704483651389	-1.70668335972379	0.81647657124977
H	3.49225355426074	0.58504920025912	-0.65679224921369
H	4.19362079921933	-0.76767351981438	-1.56895102593702
N	0.47479677172688	2.07623786063090	-1.76782810466518
C	-0.53715768515355	2.13196262006458	-2.84215610584594
H	-0.25012170921424	1.41134778107107	-3.61728679328565
H	-0.53644024114662	3.12502071742154	-3.32987695989577
C	1.83198787034092	1.95680892947920	-2.34759494205165
H	2.55180671894756	2.22497842038429	-1.56499217827150
H	1.96829331355445	2.68589507417215	-3.16847902734018
C	0.42078810912348	3.28944064863805	-0.93705692638932
C	1.24978700374666	3.15800521957082	0.35033962539297
O	1.48255539352584	1.97044667552415	0.76603664801210
O	1.61673464886434	4.21560420397142	0.91812340581513
H	-0.61844678532856	3.46653434707152	-0.62609678207969
H	0.75390453296637	4.18170218756699	-1.49774975499379
N	-2.07196349127934	0.46910472202023	-1.77614694294092
C	-2.13909982168429	-0.53492544501635	-2.85712852682490
H	-1.43018279230273	-0.23861292042949	-3.63952238929468
H	-3.13836499792909	-0.53288131507749	-3.33194472235504
C	-1.94060262516938	1.82757340341226	-2.34977067067828
H	-2.22220216749351	2.54705747379506	-1.57188533571420
H	-2.65331621006318	1.96721116235999	-3.18414280543338
C	-3.28099470128036	0.42038519919431	-0.93936952005755
C	-3.13979917922393	1.25708115317354	0.34307908891354
O	-1.94949587010576	1.47960712173212	0.75666425064108
O	-4.19315693288464	1.63882758359726	0.90912921975770
H	-3.45921421952691	-0.61781884559843	-0.62404499080049
H	-4.17596245111389	0.75268909214641	-1.49628983594566
N	-0.46120770391610	-2.08043212193493	-1.82453111475684
C	0.53864711065003	-2.10305062296108	-2.91097082818931
H	0.24296480737484	-1.35981263919916	-3.66112117113301
H	0.53355878759454	-3.08095403956045	-3.42854035306096
C	-1.82547842490590	-1.94221757018129	-2.38198590758235
H	-2.53508636131370	-2.23285361081820	-1.59788406717556

H	-1.97479959746078	-2.64659910627084	-3.22208882310060
C	-0.39894035169063	-3.31680933555688	-1.03056069780546
C	-1.20338606784901	-3.21887101120699	0.27517527022210
O	-1.41528652379774	-2.04243245039462	0.73453841737609
O	-1.57342229027522	-4.28950752879376	0.81454901644071
H	0.64442965223802	-3.50778503467073	-0.74224231295984
H	-0.74581607331663	-4.19140163453709	-1.61046788184421
O	0.02610257259640	-0.02554559443281	2.46033321733943
H	-0.06914791643906	0.88092751876261	2.79889440938641

LaFDOTA^{2-}

La	0.00941806652929	-0.02640186993535	0.11756490936373
N	2.07946271992682	-0.46560519756915	-1.80052520261593
C	2.12830234945879	0.56148589421958	-2.86137142097292
H	1.40451898074685	0.28369518279374	-3.63695208127397
H	3.11883795297015	0.56904435041036	-3.35353451640681
C	1.94775041588450	-1.81357775620118	-2.39972578504450
H	2.23371931616486	-2.54681889215710	-1.63605623482412
H	2.65839621761931	-1.93556763206307	-3.23838869001241
C	3.30083879378897	-0.42883561588097	-0.98079838390811
C	3.17648361977309	-1.27395456117939	0.29683720802204
O	1.98948906702304	-1.49250604328257	0.72749496172856
O	4.23358656338338	-1.66778174842512	0.84372872012977
H	3.48630584421910	0.60520725180015	-0.65656318482444
H	4.18627937565106	-0.75759474060306	-1.55428131115413
N	0.47008219969334	2.07803344476514	-1.76049346611237
C	-0.54547692058808	2.14536359503100	-2.83141979254298
H	-0.25936737002032	1.43449831123079	-3.61585128757189
H	-0.54729773231782	3.14401163406741	-3.30698371992298
C	1.82466356631622	1.95687261799698	-2.34701664545979
H	2.54941452047214	2.22990298642708	-1.57063773205258
H	1.95558617320281	2.68159635134808	-3.17217492071925
C	0.42458660991578	3.28429347476016	-0.91918970468543
C	1.25845408097836	3.13662083103438	0.36336810287623
O	1.46969930758976	1.94214650708282	0.77687625522538
O	1.64557252054940	4.18366349794806	0.93386866025310
H	-0.61254860803013	3.46315327482201	-0.60116283752085
H	0.75799761356710	4.18021498922831	-1.47337867152647
N	-2.07464065951527	0.46915114655147	-1.77584070413426
C	-2.13015230784991	-0.52836285025269	-2.86429866262747
H	-1.41118181514007	-0.22931608345738	-3.63644250762320
H	-3.12374206766107	-0.52251515567628	-3.35028033214712
C	-1.94657929162981	1.83358854010043	-2.33778093506773
H	-2.22812715197882	2.54515741435827	-1.55232358527283
H	-2.66197560779264	1.97891562838810	-3.16864950216669
C	-3.29020684560358	0.40911209453256	-0.94897069295509
C	-3.15628517562546	1.21754902364976	0.35121979294279
O	-1.96624914895231	1.42245008968988	0.78040481385403

O	-4.20942101618772	1.59493716525791	0.91699647068847
H	-3.47350776737217	-0.63371717673462	-0.65283072022917
H	-4.17963076111812	0.75395686103920	-1.50661417567919
N	-0.46532211298209	-2.07506472993522	-1.81563096214999
C	0.54385920897834	-2.11168439429950	-2.89403664048514
H	0.25306987247368	-1.37952565792065	-3.65690827548090
H	0.54327579515094	-3.09680052447931	-3.39699913498941
C	-1.82346660471617	-1.93745427818241	-2.39036629448700
H	-2.54352711051977	-2.23186850303569	-1.61745139698136
H	-1.95965578350384	-2.63898195533910	-3.23450864079248
C	-0.41357844337552	-3.30528423979952	-1.01008709390335
C	-1.23521393656570	-3.19520010057669	0.28398986122669
O	-1.44613677222615	-2.01306448672974	0.73146915404385
O	-1.61603922387203	-4.25855170967513	0.82796961804627
H	0.62618666512925	-3.49381328725397	-0.70656271805985
H	-0.75242835166804	-4.18459533348213	-1.58710762564034
F	0.01694116965626	-0.05873763440664	2.39869766162357

LaDOTAM^{3+}

N	2.08091596893917	-0.43123151005389	-1.78219683156654
C	2.11915295569604	0.61838250050335	-2.83317170525942
H	1.41599436174364	0.32718729270470	-3.62126432722514
H	3.11480692508251	0.65672585064780	-3.30479215571361
C	1.99911509240165	-1.78324191686146	-2.40233948093955
H	2.30985897041041	-2.51811708982643	-1.64947591646018
H	2.71723217618001	-1.86497395640541	-3.23458157609827
C	3.29269666901456	-0.36072053221990	-0.94461271351968
C	3.10577625674381	-1.18561654033317	0.31513423060560
O	1.94937739399326	-1.33518075579620	0.80096725777608
N	4.18343808540769	-1.70125152350590	0.87826336206220
H	3.45294100285735	0.67683588072771	-0.61906905244353
H	4.19271384131544	-0.67290659262431	-1.49867556555825
N	0.40010775262083	2.06393398887833	-1.72267203544486
C	-0.61643958925298	2.12767742422577	-2.80462233010476
H	-0.30112939815620	1.44292836205749	-3.59958194421820
H	-0.64005461397081	3.13419677442833	-3.25365277546304
C	1.77108825932140	1.99609401173345	-2.30184380745763
H	2.48188656574620	2.28946920340351	-1.51949229360661
H	1.87849417018009	2.73304198515108	-3.11451523815678
C	0.30288166400396	3.25481296628367	-0.85829709686526
C	1.08732659813782	3.03729963311042	0.42222903824604
O	1.22494366101992	1.86925845529019	0.88328973834880
N	1.58143486158761	4.10112196744229	1.02917975348700
H	-0.74444281031028	3.40697844492012	-0.56158593230199
H	0.63168288135404	4.16799418907109	-1.38020137999032
N	-2.09466321993608	0.38475461377107	-1.77789605219917
C	-2.12537226722790	-0.60721794448065	-2.88377620830528
H	-1.41601810052866	-0.27508740053858	-3.64982088380634

H	-3.11736290089718	-0.61955011716633	-3.36451724147776
C	-2.00991673733542	1.76815549209169	-2.32452227406898
H	-2.32835654371470	2.46178672071122	-1.53661240738627
H	-2.72106091250630	1.89326451972987	-3.15740816958830
C	-3.31096449562709	0.26846675221610	-0.95201562201611
C	-3.13153490941434	1.02402342543596	0.35149313904687
O	-1.97781889938369	1.14925240235253	0.85056783707012
N	-4.21265517695531	1.50593839556844	0.93740468865933
H	-3.47174910266583	-0.78510464077780	-0.68292022827013
H	-4.20830855586253	0.60912014885435	-1.49336688068057
N	-0.41565598140427	-2.10997305174107	-1.83732730954501
C	0.60959613847699	-2.11497887762696	-2.91278624961759
H	0.30086474865831	-1.38769364575300	-3.67174168175537
H	0.63688946083747	-3.09538799192054	-3.41609373167942
C	-1.78164052897889	-2.01143570336097	-2.42389227040964
H	-2.49909700562816	-2.34622484576097	-1.66458448300423
H	-1.88185208783696	-2.70395418444732	-3.27563490109203
C	-0.32421202799924	-3.34600015440648	-1.03813531850843
C	-1.12010290745377	-3.19787605751452	0.24518921628616
O	-1.25908464163452	-2.05652837033057	0.76842235438934
N	-1.62234329553934	-4.29229571200050	0.78777724369216
H	0.72071249793714	-3.51355787891902	-0.74114148667718
H	-0.64762838903433	-4.22991078333777	-1.61116186146658
La	-0.01178767844010	-0.07107270762664	-0.05074953011584
H	1.48242419848012	5.02930255423664	0.63277117124062
H	2.05433978257256	4.00319993957886	1.92319487474804
H	-5.12920356888999	1.41268501015673	0.51360501853777
H	-4.14161715122430	1.95489528394684	1.84620038521539
H	-1.51820884789308	-5.19761341797352	0.34281908435456
H	-2.10209996314646	-4.24359726138543	1.68221918642730
H	5.10117766613265	-1.59010941050236	0.46134522746781
H	4.10829170199606	-2.20058361403189	1.76006614240266

La(H_2O)DOTAM^{3+}

N	2.06262180764639	-0.51052398595467	-1.76041056257355
C	2.13784101510761	0.53817543862433	-2.80859111977180
H	1.41878390698809	0.27747371922000	-3.59301810804708
H	3.13100474781610	0.53629179067683	-3.28803654820762
C	1.91703047256369	-1.85489911075877	-2.38437774067815
H	2.20702916307706	-2.60584265210809	-1.63938280594605
H	2.61999264044382	-1.96285820036871	-3.22687808145961
C	3.28503566842378	-0.49813808061447	-0.93710552810494
C	3.08113358377435	-1.35536100526296	0.29818112934884
O	1.92618961977447	-1.49185782623321	0.78280693553371
N	4.15039355047728	-1.91825897586743	0.83775767928770
H	3.48221055763308	0.52489109680330	-0.58674052961375
H	4.16875922936033	-0.82534208133980	-1.50951333680889

N	0.48595333363856	2.05039812774344	-1.68806265104330
C	-0.52681918387168	2.14609139838088	-2.77055834011848
H	-0.23827944260708	1.44182725070846	-3.55850970653785
H	-0.50780060640727	3.14833301021742	-3.23041921207974
C	1.85063243261097	1.92690537391835	-2.27132191358690
H	2.57551845555838	2.18396792905251	-1.48908386014352
H	1.98797072403314	2.66389140051678	-3.07986520762065
C	0.43958108764002	3.24904469197628	-0.83213834226967
C	1.23653623019702	3.01539473345458	0.43895947140297
O	1.36293850453840	1.84738462153725	0.89884313630306
N	1.75586275236264	4.07320785196892	1.03844556334275
H	-0.59796981018273	3.43919705234285	-0.52322043003460
H	0.79360582021341	4.14753878389722	-1.36402288743070
N	-2.08154102582323	0.47543808388188	-1.73550959056465
C	-2.15639095284476	-0.52367559678294	-2.83093833547503
H	-1.43805319274529	-0.22693703246371	-3.60308704779415
H	-3.14978714156837	-0.50084772312098	-3.30928688884313
C	-1.93497017562818	1.84875579526722	-2.29314611859635
H	-2.22400327250979	2.56204638816297	-1.51171967946102
H	-2.63833825360867	1.99844001393631	-3.12895122091658
C	-3.30382518674402	0.42393114024161	-0.91418039719438
C	-3.09725513691544	1.21312794332443	0.36512886609257
O	-1.93983848262635	1.33109212818010	0.84893572677601
N	-4.16662873702263	1.73757587763282	0.94149164878893
H	-3.50570908119759	-0.61540289623541	-0.61879819598999
H	-4.18638065509561	0.78417371763774	-1.46830321148540
N	-0.50122606879320	-2.08693249974812	-1.78551276690605
C	0.50853098995844	-2.12872351025410	-2.87431208695410
H	0.21847001294266	-1.38734132949218	-3.62687116334113
H	0.48886750795805	-3.10751316095823	-3.38193065711999
C	-1.86800651986346	-1.93545596236540	-2.35744518231186
H	-2.58971287546782	-2.22708048346811	-1.58421147458634
H	-2.00952289526451	-2.63532569493027	-3.19762247193149
C	-0.45011856415992	-3.32887806734064	-0.99377432799822
C	-1.23224252261319	-3.16302048484462	0.29634678156757
O	-1.34007370889321	-2.02349786059036	0.82727152999950
N	-1.75652752028990	-4.24926888765030	0.83795248595844
H	0.58983508803744	-3.53592315612435	-0.70468417489677
H	-0.81010744059120	-4.19755938758651	-1.56929936950193
La	-0.00699508927571	-0.05902856430929	0.02028087373871
H	1.66565619504561	5.00234516495346	0.64231391039814
H	2.23670968437335	3.96877344213689	1.92732079031994
H	-5.08712646540757	1.65138123945672	0.52505515073373
H	-4.07979955562324	2.21882224615106	1.83198620838251
H	-1.67981824594722	-5.15421469965967	0.38676744929113
H	-2.22863622209929	-4.19330144699463	1.73590340329908
H	5.06848204121437	-1.81723746971941	0.41940459919742
H	4.06544020325792	-2.44662851982652	1.70133101641915

O	0.00706978817141	-0.10772113859807	2.65222816509675
H	0.78509772211100	0.30593589844612	3.06879985468100
H	-0.04506050526074	-0.99947585887683	3.04165889798577

La(OH)DOTAM^{2+}

N	2.08063016497366	-0.51163977583582	-1.71209934211417
C	2.11877234184173	0.52381263944194	-2.76993610389406
H	1.37808680167258	0.25336456889995	-3.53066078496707
H	3.09783650289110	0.52222858277774	-3.28122805026790
C	1.93735628440194	-1.85971153416492	-2.31940326001734
H	2.22244999718928	-2.60347786539087	-1.56541306606420
H	2.64306247092130	-1.98021589715498	-3.16048385945522
C	3.31638117931767	-0.47582598761987	-0.91871252682075
C	3.16566588478067	-1.34960840991356	0.31436432098177
O	2.03289524774310	-1.53826843194421	0.82210775741232
N	4.27542946320714	-1.87062346351352	0.82525140578122
H	3.49474700909003	0.54887376592600	-0.56261687573126
H	4.19880451799001	-0.77714138789599	-1.51037833846616
N	0.49129825916033	2.05869240253704	-1.64424875884571
C	-0.53486196595861	2.15030797330377	-2.70791726387803
H	-0.25383657356182	1.45260502915587	-3.50448409508029
H	-0.53170709285185	3.15505427418120	-3.16637640827501
C	1.84049773510845	1.92234004814063	-2.25118587726608
H	2.58471605834125	2.19251364391308	-1.49251564049020
H	1.96404536607770	2.64174009857460	-3.07983058959213
C	0.46235711486439	3.26182382564563	-0.80154216626511
C	1.33809030102467	3.05704180718094	0.42316018652599
O	1.52159171607356	1.90355740358646	0.88321952666428
N	1.86701782363508	4.14247417195068	0.97674480052422
H	-0.56191778977758	3.43102411387411	-0.43877866576553
H	0.76733881200402	4.16628537996606	-1.35699993818398
N	-2.07692299839718	0.46561164901961	-1.67560857525298
C	-2.12152614743896	-0.51526354789117	-2.78468499585167
H	-1.38464279070772	-0.20698516085427	-3.53455038002107
H	-3.10335283639262	-0.48678293252898	-3.28952156060255
C	-1.93727053015568	1.84203921535706	-2.21834732639273
H	-2.21844267506337	2.54834520395772	-1.42791188809534
H	-2.64799417493082	2.00140356525389	-3.04854679920236
C	-3.31197025842875	0.39133175679719	-0.88211341402466
C	-3.16097733228550	1.20811884125294	0.38937888434300
O	-2.02710999452141	1.38063092723819	0.89924888774881
N	-4.27136657478575	1.69818145256905	0.92956402318496
H	-3.48830942728454	-0.64908626740655	-0.57400425643571
H	-4.19521200564387	0.71858978414804	-1.45843886403478
N	-0.48980586547435	-2.10204647285610	-1.74351791645823
C	0.53194171241115	-2.14365317675802	-2.81517352652261
H	0.24759721082031	-1.40918863974864	-3.57681252556043

H	0.52543413819815	-3.12581278174882	-3.32012256611205
C	-1.84134166441190	-1.93801149582089	-2.33855364974546
H	-2.58270250885449	-2.24643917940687	-1.59199339025116
H	-1.96668077456713	-2.61496453894355	-3.20187737806249
C	-0.45797465908218	-3.34388528660668	-0.95846339080436
C	-1.33959441740507	-3.20006444819000	0.27106427384997
O	-1.53503010435539	-2.07025117497761	0.78150390249757
N	-1.86166304529853	-4.31368147810454	0.77361444509882
H	0.56602523843922	-3.52641802883915	-0.60136937063258
H	-0.75808163143316	-4.22199135042069	-1.55720608078461
La	-0.00145081941582	-0.06917003102972	0.20808699673739
H	1.72478901405231	5.06245573497805	0.57548364921023
H	2.40624183880373	4.06451689321732	1.83375206855426
H	-5.17525307062141	1.56708842067733	0.48991721558847
H	-4.22883784296106	2.19666402203561	1.81324971369605
H	-1.70940058505323	-5.21387859351123	0.33328149299480
H	-2.40419892383689	-4.27836155639937	1.63131445526417
H	5.17774963716360	-1.72351434990420	0.38727886497513
H	4.23565304839654	-2.40679126180128	1.68677643007006
O	-0.02016689160263	-0.13581908617818	2.47461540972803
H	-0.88643891803535	-0.22471360219746	2.90660075485648

LaFDOTAM^{2+}

N	2.07466999117689	-0.50477711029888	-1.70728279757717
C	2.13081727986299	0.53566100924331	-2.76094738684758
H	1.40539594085334	0.26583764248075	-3.53635297877929
H	3.11921324935536	0.53748829847043	-3.25292770179713
C	1.93329294628607	-1.84896473387141	-2.32617269633620
H	2.22107484977387	-2.59896473084352	-1.57959885795626
H	2.63797881210285	-1.95984163926428	-3.16872187211767
C	3.30281536540612	-0.47873424293950	-0.89926187031291
C	3.13533570193034	-1.36414989648276	0.32330316492801
O	1.99505684534258	-1.54851935593145	0.81757368648830
N	4.23426751793182	-1.90028822609491	0.83926168523644
H	3.47981888826190	0.54257816948633	-0.53261383094299
H	4.19014498734168	-0.77662857734099	-1.48452961821989
N	0.48228126760313	2.05826014296215	-1.64932281249677
C	-0.53394454316387	2.13750785962563	-2.72488130606621
H	-0.24691147392233	1.42804068866911	-3.50876832596131
H	-0.52420691382364	3.13596690848122	-3.19607118447994
C	1.84031557116884	1.93029990605632	-2.24030469813450
H	2.57282622222795	2.20031547611766	-1.47014939996853
H	1.97040239944214	2.65413930549919	-3.06366000337989
C	0.43758434187698	3.26861105404071	-0.81589462348815
C	1.29070476164916	3.07369988467387	0.42537430523010
O	1.46839071632963	1.92210933817567	0.89508684543954
N	1.80593297772104	4.16187086162871	0.98384397179542

H	-0.59253296760205	3.44018103760614	-0.47193728379030
H	0.75182190885334	4.16769549134150	-1.37406632080747
N	-2.08164406370726	0.46626314933474	-1.68445005201019
C	-2.13804596255730	-0.52598131643588	-2.78361594877553
H	-1.41314876187961	-0.22176128062816	-3.54667626515076
H	-3.12671225968207	-0.50617526442023	-3.27461786486364
C	-1.94010608496919	1.83698170152658	-2.24222773521945
H	-2.22730872950395	2.55256791910715	-1.46243868903194
H	-2.64516381321569	1.98583965230809	-3.07861136139315
C	-3.30994543707772	0.40409288122300	-0.87889539931281
C	-3.14177425667965	1.22857976005802	0.38539912501412
O	-2.00075073657004	1.39341970162597	0.88465974294954
N	-4.24135953498824	1.73315407358949	0.93102464316636
H	-3.48973803703645	-0.63332778765395	-0.56224316427940
H	-4.19635189038703	0.73196187419683	-1.44930573327612
N	-0.48851105467272	-2.09645320179894	-1.74193082346186
C	0.52690085739301	-2.12812306701224	-2.82075237871935
H	0.23925784325820	-1.38541972429481	-3.57308258023607
H	0.51716717462463	-3.10513333171999	-3.33485843602362
C	-1.84699228591903	-1.94234264867708	-2.32557834275176
H	-2.57873814395367	-2.24617014863015	-1.56737147906869
H	-1.97796337857610	-2.62916698967136	-3.17991939903283
C	-0.44341106307429	-3.34284946352626	-0.96341752682223
C	-1.29609721588437	-3.20374452403027	0.28575691124968
O	-1.47056028221944	-2.07480443531726	0.80878833969045
N	-1.81357816248909	-4.31527741216305	0.79369197076532
H	0.58681704732327	-3.52921172968377	-0.62773803805953
H	-0.75765783434101	-4.21621016102468	-1.56109799039613
La	-0.00241787684315	-0.06118380278201	0.18154720494742
H	1.67010508261125	5.07973397021724	0.57545491370825
H	2.32804355469829	4.08905584925995	1.85195526156169
H	-5.15020370177150	1.60780922856263	0.49973893265066
H	-4.18698532533604	2.23624336600456	1.81161099066858
H	-1.67848581606310	-5.21389431197017	0.34434511905649
H	-2.33523440105290	-4.28140589146633	1.66445876408992
H	5.14233607501325	-1.75754943204909	0.41171947558024
H	4.18042582487882	-2.44687890406795	1.69358638344285
F	-0.00085399333623	-0.10891285948165	2.40829333968583

LaDOTPy^{3+}

La	11.07656347899940	5.56710235787422	5.11781249250044
C	13.12653651625666	3.64345320069247	2.93214061123983
C	9.02070326832890	7.50445872106310	2.94928417399623
H	10.00746955542106	7.64567825583251	2.50687851433960
C	13.16540039018465	3.87603037605873	7.32391425500396
C	8.99428565372046	7.24669529791250	7.33915385981415
H	13.51412312294928	3.17923682002138	6.55249541195072

H	8.64320997594832	7.94775029695430	6.57268154969004
H	13.88265230838427	3.80592489047386	8.15801366697501
H	8.27971233303119	7.31221865580051	8.17584662586953
C	11.78879053099028	3.46793408776203	7.81777442903591
C	10.37237717528533	7.65200705844609	7.83107019282500
N	10.76336483811538	3.45446650351581	6.73937891218339
N	11.39466188380465	7.67113919276667	6.74979049466725
N	13.18725976198444	5.24731227134038	6.74066980854997
N	8.97057268797448	5.87868924149474	6.74846492835581
C	14.40658328717128	5.40805454071504	5.91154034261546
C	7.74891881741035	5.72249913701958	5.92182705303538
H	14.48577795968452	6.46307523651819	5.61194039171830
H	7.66932438136901	4.66927339485715	5.61605235182070
H	15.30847941480123	5.17781676134006	6.50151669744312
H	6.84852405457222	5.94879754790602	6.51569573732578
C	9.52902366332571	1.09274662999215	4.13648386611139
C	12.62027179681300	10.04655353938346	4.15499303307840
H	12.46023601433297	10.99155488920595	4.67111329292343
C	14.38050266859261	4.55805594720534	4.66698363245368
C	7.77154831422956	6.58008760521699	4.68240035154586
N	13.18065849479417	4.36467851385965	4.07143341182251
N	8.96962693441277	6.77597614452589	4.08408394676260
C	10.92019898089466	2.23592020862960	5.90825617000613
C	11.23525500755664	8.89381984988575	5.92531087758515
H	10.69275468200891	1.33336132327521	6.49843360589470
H	11.46448352492781	9.79333704536566	6.51931795776314
H	11.97376847266021	2.15709579849933	5.60348858015221
H	10.18075029404778	8.97420766511225	5.62420631985408
C	8.79270081503308	1.15622972639126	2.95602575257425
C	13.35332199022911	9.98936261042302	2.97219208340162
N	9.86957357995426	3.46413695101968	4.07392926575212
N	12.28092871136800	7.67528120771795	4.08162929431824
C	15.55255995026938	4.02757985981841	4.13333908306273
C	6.59826969401713	7.11508299577470	4.15601782138681
H	16.49972917274983	4.18741998031329	4.64553381742291
C	8.59427117671744	2.39248709003775	2.34298571577251
C	13.55094948289419	8.75624063328911	2.35258302543495
H	8.01942286503235	2.48743623500580	1.42442672780276
C	9.14252486502530	3.51991235263543	2.93844541726385
C	13.00432923324501	7.62565991411949	2.94351581096289
H	9.00345177419474	4.50828790988000	2.49896255911770
C	14.25501166476662	3.09944095136129	2.33480866919741
C	7.89088736640918	8.05390616159189	2.35948471119793
H	7.98218933467907	8.63100094165393	1.44196296605491
C	12.76503942244365	7.64680268949874	7.33501567635442
C	9.39460160508925	3.47598679454666	7.32845471203554
H	13.46301452227889	7.99889354998819	6.56620318512873
H	8.69449937703563	3.12808966370471	6.55966340614252

H	12.83381163658512	8.36042208507225	8.17231442924064
H	9.32794864230475	2.75798942480755	8.16209496098967
C	13.17241002068750	6.26808335577741	7.82348593088140
C	8.98861630449974	4.85201234321709	7.82556712464085
C	15.49075172593513	3.29714576847775	2.94916250710326
C	6.65695200037465	7.85330548018541	2.97650002931044
C	10.06413398746285	2.26368568697315	4.66789923201090
C	12.08752319018471	8.87262099639323	4.68221012155667
H	11.85777937511163	2.47425277786539	8.29025015657305
H	11.44527597698081	4.15618495808501	8.59820362052314
H	10.30490988490420	8.64313476760421	8.30908564590160
H	10.71811090021371	6.95953190957436	8.60676402087718
H	12.48308197763364	5.92125712716777	8.60148966875363
H	14.16545942500918	6.33495683999650	8.29757497960215
H	7.99694804814815	4.78241116581704	8.30217184306879
H	9.68014941182521	5.19461857454408	8.60348838881490
H	13.14354506120887	6.63945148076922	2.49924728730418
H	14.12384770376799	8.66612730400803	1.43232164784168
H	13.77488993515336	10.89761061210251	2.54409343512472
H	16.39750667459314	2.87766658402658	2.51587886358935
H	14.16135469940265	2.52868355470652	1.41357288363794
H	12.13839994139786	3.50392003965652	2.49228487453971
H	5.74929066378440	8.27702690673467	2.54928208276657
H	5.65263172436666	6.95288623669057	4.67029459644373
H	8.36973189011365	0.25031239191438	2.52438160678098
H	9.69019234993415	0.14505440253894	4.64729281482918

La(H_2O)DOTPy^{3+}

La	11.07486242399389	5.56555913770354	4.97813783001354
O	11.06940062174709	5.56980971452092	2.33451834999635
C	13.24341073634480	3.42513249558474	3.02542189023898
C	8.89980532815029	7.71690525737310	3.04384591591580
H	9.87122974539153	7.91117012185428	2.59078003000480
C	13.17673604821500	3.89992335576836	7.27508274116479
C	8.98130908281442	7.22167995825734	7.29255804063695
H	13.54532580461080	3.19972738145548	6.51708198242679
H	8.60861706937665	7.92583996835442	6.54027486295081
H	13.88295985319288	3.84805994207230	8.12033549242365
H	8.27943883331865	7.26881870605245	8.14183002064441
C	11.80174407985048	3.47882553877262	7.75210344294158
C	10.35859749398526	7.64078165177178	7.76492630136076
N	10.78371248556820	3.46039442429625	6.66806863661465
N	11.37205029964493	7.66329227638434	6.67672456376024
N	13.18465071696598	5.26673147280147	6.68237872070567
N	8.97109973145873	5.85811773038984	6.69264499918941
C	14.41821535825680	5.43564711718007	5.87900299819763
C	7.73495159270244	5.69362990241854	5.89202110014768

H	14.47067149085428	6.47818285502449	5.53483206892871
H	7.68282934655034	4.65361951563188	5.54023931942157
H	15.31295919469710	5.25981008251816	6.49939125664813
H	6.84205830442396	5.86376233127018	6.51644861234993
C	9.55473160874876	1.03518620380587	4.12052194522905
C	12.58823600457897	10.09902900532573	4.13223965817697
H	12.31371661937769	11.03974825714006	4.60658360402060
C	14.44187115901869	4.51990564675800	4.68460220802994
C	7.70715709693680	6.61835359710593	4.70474133360723
N	13.25686282754479	4.25179718876235	4.09119622391862
N	8.88924448669249	6.88303459254170	4.10385776309233
C	10.95355259275897	2.24313671847236	5.84017945898131
C	11.19903299842536	8.88394870999081	5.85444191172997
H	10.77985089231524	1.33536118837608	6.44200969290721
H	11.37561880771551	9.78917123119858	6.45916774653265
H	11.99695754185958	2.19819984063729	5.49652856122255
H	10.15422280658465	8.93048221075094	5.51530381616962
C	8.71354866034103	1.06610491535899	3.01250137590696
C	13.42429357948235	10.07292026380911	3.02020029768911
N	9.71371072742939	3.42854278351734	4.09933420424762
N	12.43270422232162	7.70562349404929	4.10385756691847
C	15.63904002443963	3.98430292819546	4.21313085611440
C	6.51012314741456	7.16622629865236	4.24747099107668
H	16.57243558020201	4.21920787457488	4.72201419095298
C	8.35355007400787	2.29885695651785	2.46860357085959
C	13.78209890319383	8.84259742896078	2.46939315613292
H	7.67840058598980	2.37301471514265	1.61885094479465
C	8.87172466232078	3.44951985711903	3.04492215480356
C	13.26827499273334	7.68929418891712	3.04430806451077
H	8.59428012926741	4.43472005332711	2.66563536157560
C	14.39357271643531	2.85074846043237	2.50177804092658
C	7.74938986723441	8.30339791410496	2.53405058738572
H	7.81506177338024	8.97286734880617	1.67892377439934
C	12.73786860973992	7.65893596385317	7.27276943071874
C	9.42012905751005	3.46259279894932	7.26944451553835
H	13.44203931234625	8.01724194757725	6.51389730463340
H	8.71288349446538	3.10821156192062	6.51162420296953
H	12.78751536675731	8.37323894488865	8.11119394062570
H	9.37342187049966	2.74431750817276	8.10466812396652
C	13.15034302516180	6.28523242807754	7.76428170666756
C	9.00949570850877	4.83396770683405	7.76907663475880
C	15.61637367827806	3.14438413942740	3.10330958011254
C	6.52959401603669	8.01424068351058	3.14371782902090
C	10.03842803517963	2.23405321064234	4.64300658195150
C	12.10864359740861	8.89779036954912	4.65294934761414
H	11.87302155722456	2.48370327437859	8.22236361855455
H	11.44688576762006	4.16157901465769	8.53194380413423
H	10.28933927095060	8.63406501432719	8.23936300912854

H	10.71662895226493	6.95495882478222	8.54058671886990
H	12.45526957329731	5.93358492481754	8.53478777406393
H	14.13802690726145	6.35807463610849	8.24981894247611
H	8.02353879988178	4.75827470557439	8.25768666542275
H	9.70717624721020	5.18197933464686	8.53888396084267
H	10.50435429097251	5.04415931748887	1.74172869607892
H	13.54411327291921	6.70574843511359	2.65956733033619
H	14.45217872046180	8.77239438306634	1.61531723616168
H	13.80747755179180	11.00117821500392	2.59903756179421
H	16.54093023887637	2.71453588275660	2.72036926977381
H	14.32565347157474	2.18742945977714	1.64203239689624
H	12.26964222667985	3.22729177690644	2.57901393646066
H	5.60523121729728	8.45463204332317	2.77246749676861
H	5.57944915801244	6.93420452115154	4.76261155144769
H	8.32861198987091	0.13966783966178	2.58896624789988
H	9.83153020613354	0.09246769706244	4.58955173352947
H	11.63467407694097	6.09479262618475	1.74124461218648

La(OH)DOTPy^{2+}

La	11.09779290719765	5.54764047373895	4.78551339704643
O	11.17116120732864	5.56341067275132	2.53514677398550
C	13.35712980410965	3.27745611758693	3.04189495046476
C	8.84398098193869	7.63878305690642	2.93532663810681
H	9.81268113637701	7.75813713489558	2.44942202618149
C	13.16564204827492	3.87188606791742	7.22697510244888
C	9.01011768092472	7.23314917670747	7.22028205222643
H	13.51982288887098	3.16004022683838	6.47350609933793
H	8.66706569817123	7.94290018136331	6.45834119062941
H	13.86645895569269	3.80483837175473	8.07800497492148
H	8.29969335291181	7.31001069610396	8.06268921611748
C	11.78056723353678	3.47536173394725	7.70295671288678
C	10.39186890817659	7.62534016450731	7.70858390112449
N	10.76606936975628	3.43519027520873	6.62166291799620
N	11.40901789001111	7.67736627497638	6.63132501044101
N	13.20831219839888	5.23288322656499	6.63103410237985
N	8.96239399270610	5.87079281849082	6.62917650531619
C	14.45440119674914	5.38502284545163	5.85340877575594
C	7.70706390608520	5.72601446342554	5.86320141859964
H	14.51739767290580	6.42398562295829	5.49666875429007
H	7.62789610399503	4.68381985203499	5.52031586462196
H	15.34081491559717	5.21695720921275	6.49111581169624
H	6.82879072751591	5.91817427794463	6.50478269301282
C	9.44686779859512	0.94060330741983	4.20631594714058
C	12.74902168051808	10.18499290959141	4.25142542067376
H	12.55139571822239	11.10402336984520	4.80151436498881
C	14.50999126295404	4.45870796415920	4.66487011143691
C	7.66658120681064	6.63392773206476	4.66172921589431

N	13.33743200769614	4.10383198453295	4.10407528051408
N	8.83579783617458	6.85379753206924	4.02868089203416
C	10.91712461635484	2.18822554638734	5.84185782175103
C	11.25773203771421	8.93332416976547	5.86705539227802
H	10.74205839143886	1.30184085235550	6.47704338412790
H	11.43495096877840	9.81188529935770	6.51242833155072
H	11.95624998972234	2.12579051034618	5.48727238285945
H	10.21783075662121	9.00212339494231	5.51483199856560
C	8.60427097119167	0.94776608756500	3.09822505792973
C	13.57646981181515	10.18047187501112	3.13123335081797
N	9.72970134237300	3.31659080395142	4.03733467157296
N	12.39795537665453	7.82285892963518	4.02884546670756
C	15.73473178482577	4.00929851370135	4.16691845992291
C	6.46952231588509	7.19554732775593	4.21631599762587
H	16.66152441317882	4.31643138301067	4.64990825289083
C	8.32043405007806	2.16070051278771	2.47096925128374
C	13.81028611363924	8.97700944749352	2.46672480474239
H	7.65726049152365	2.21421496967819	1.60961025099702
C	8.90621349433176	3.31699395746218	2.97156294608637
C	13.19623367022689	7.82507869719445	2.94510632380245
H	8.72453646653577	4.28906888366130	2.51070745040481
C	14.53138707222089	2.77955091168034	2.49237921097297
C	7.69576230590356	8.24121018996275	2.43452981532029
H	7.75312503758139	8.87485508355271	1.55122903988561
C	12.77107007995295	7.63314917189872	7.22324812533719
C	9.40370236054231	3.47342837076121	7.21509837623760
H	13.48060069769209	7.98041163968569	6.46302127892746
H	8.69371474024656	3.12963721807496	6.45413109885135
H	12.84550616565067	8.34140533957596	8.06770766680141
H	9.33245281229680	2.76060553022529	8.05572701105353
C	13.16649671591679	6.25089774200555	7.70808149506539
C	9.00752460366521	4.85310580338476	7.70582183495412
C	15.74571409138669	3.16295433827771	3.06283747407515
C	6.48476443091003	8.00958226983159	3.08665889855224
C	9.99211032001769	2.14512573179532	4.65247436951017
C	12.17661723255526	8.98534706560785	4.67458281565470
H	11.84553483643728	2.49262519596284	8.20311354359729
H	11.43063486845388	4.18089097570915	8.46447959784687
H	10.32345325453682	8.60317147746782	8.21798384513723
H	10.73791979517229	6.91289504801054	8.46538767090069
H	12.45997485333831	5.90459000174983	8.47037930947209
H	14.14849174262877	6.31878672191440	8.20946588906982
H	8.02691460517981	4.78281325645299	8.20956512022637
H	9.71538931233787	5.19821624136487	8.46731916435875
H	10.99918269010495	4.82132216833569	1.93547676418161
H	13.31332809185826	6.86136469429510	2.44836649342976
H	14.45463549272785	8.92785875624178	1.59069570953468
H	14.03843565606607	11.10472346057784	2.78545951599519

H	16.68912650727708	2.79782193325699	2.65829007165233
H	14.49178559474787	2.10771374113692	1.63692461030773
H	12.38661980848549	3.01269449941268	2.62099670750722
H	5.56296019629800	8.46273979794449	2.72320458233505
H	5.54425128472882	6.99963907718242	4.75651857815646
H	8.16541466219294	0.01908659293756	2.73509104372325
H	9.67669272979651	0.01476305069285	4.73186555318063

LaFDOTPy^{2+}

La	11.08869366245193	5.55285035445206	4.82377873447473
F	11.08813782051825	5.55298026234736	2.60707946098813
C	13.31714436624478	3.45581274318621	2.94164593068923
C	8.85926975995344	7.65019124282443	2.94310439508871
H	12.34706713894486	3.31842651165574	2.46336881301308
H	9.82901403619268	7.78726314601293	2.46406255399809
C	13.16311710287831	3.86377331310727	7.23039831970305
C	9.01552184895477	7.24166417753076	7.23167088331292
H	13.50588187233975	3.15448588834263	6.46799615195168
H	8.67234260664696	7.95104208199719	6.46953931228360
H	13.87437327040872	3.78844550398574	8.07179346710640
H	8.30471828794481	7.31688587230068	8.07346098235978
C	11.78119703514382	3.47193629076025	7.71898545115491
C	10.39770309842711	7.63344810186942	7.71956821453688
N	10.76348989096860	3.43145609109704	6.64082010836353
N	11.41484458173984	7.67405209756671	6.64087914623495
N	13.20842367343468	5.22709568476743	6.63905669230037
N	8.96990019390482	5.87841293129805	6.64018633883322
C	14.46071146811468	5.37303747873577	5.86587453364010
C	7.71720603768630	5.73256096600885	5.86765612151120
H	14.53712648522535	6.41516308271468	5.52210752682702
H	7.64058160353202	4.69046592469474	5.52384095631856
H	15.34148295028654	5.18263854320031	6.50409298075909
H	6.83677736202025	5.92292319736242	6.50635742987281
C	9.44975039857427	0.94411278344805	4.21171074859359
C	12.72751571666740	10.16159607742935	4.21144200174549
H	9.64764969803303	0.01755266702563	4.74884087634375
H	12.52988876977006	11.08810808584381	4.74875463132862
C	14.49806228034423	4.46545159409981	4.66371335772218
C	7.67922838857130	6.64022787336356	4.66558792633388
N	13.32581243445373	4.23762377272301	4.03752183526588
N	8.85117209277745	6.86818108705075	4.03884306745353
C	10.90883691003756	2.17863987167925	5.86878661170141
C	11.26912058826584	8.92694441685514	5.86903930447618
H	10.71662720536366	1.29840111360626	6.50728373101178
H	11.46163096024894	9.80712044727826	6.50753152639127
H	11.95124758657297	2.10087396357433	5.52628644843510
H	10.22655047851011	9.00474328228126	5.52703146274698

C	8.64248385447666	0.95984082937165	3.07720965140037
C	13.53424104675014	10.14596413358595	3.07655349813096
H	8.19675385923195	0.03716859000214	2.70703543739473
H	13.97979233047800	11.06866767660999	2.70624198136067
N	9.77754194718329	3.31333442526936	4.03771297690558
N	12.39951687991331	7.79241350526451	4.03731576763037
C	15.69549049346770	3.91265621924278	4.21003818371232
C	6.48155835696206	7.19299316573697	4.21253468128520
H	16.62235944955646	4.11248026741490	4.74590597455140
C	8.40732600711159	2.17208780092957	2.42922328664561
C	13.76899876273086	8.93378832441159	2.42829099584441
H	7.77858994473730	2.23004050000796	1.54258213335224
H	14.39720756799405	8.87592412300065	1.54127075925806
C	8.99745533285166	3.32180970501894	2.94051193244630
C	13.17911066058394	7.78402441951976	2.93976529135629
H	8.86254890702688	4.29153786385151	2.46082350430087
H	13.31366091291611	6.81436385520414	2.45983975608387
C	14.46693397055341	2.86550710986504	2.43067124370851
C	7.70910154185052	8.23998834236906	2.43239319269950
H	14.40877025326403	2.23477374585802	1.54546921337947
H	7.76671876300753	8.87040343236032	1.54692771650477
C	12.77837469827784	7.62694608571012	7.23114301415061
C	9.40025879509081	3.47849393730155	7.23178038816853
H	13.48737226239571	7.96995926989159	6.46853611357175
H	8.69087638887945	3.13557129222305	6.46949036293705
H	12.85521277829993	8.33719999425890	8.07327880967775
H	9.32384645736849	2.76813932034543	8.07386985388723
C	13.16917448493653	6.24419392827665	7.71804166521317
C	9.00971627611452	4.86119033457043	7.71903674645087
C	15.67955246019671	3.10331359920522	3.07697724097377
C	6.49684845582063	8.00227304931718	3.07942876899894
H	16.60236532898132	2.65788610613750	2.70678533650104
H	5.57381119743396	8.44753214533857	2.70961059642767
C	10.00293446839493	2.14140513436027	4.66536470892813
C	12.17446519785875	8.96428344850105	4.66520302548985
H	11.84720540726912	2.49019687178952	8.22053570419325
H	11.43753462432808	4.17977119052075	8.48122844139515
H	10.33196398716823	8.61512565189132	8.22127522936928
H	10.74176920384298	6.92551572117516	8.48153654571154
H	12.46104761516741	5.90041154098513	8.47995458190720
H	14.15108982740343	6.30861115706256	8.21941520942945
H	8.02806818382375	4.79672038746730	8.22092627042099
H	9.71824786352749	5.20488678825585	8.48061094464867
H	5.55498353261862	6.99298648644289	4.74881522872493

LaMacropa^{+}

La	-0.00009691637864	0.57409511469031	-0.00015327971335

O	-0.33029783872947	-0.62560982030118	2.10028324544862
O	0.33002974818397	-0.62670278063228	-2.10025098356976
O	-1.72725295436999	1.97543235528319	-1.51892687192563
O	-0.99427164900245	2.26596863173375	1.99709749759441
O	1.72694752985920	1.97526299103298	1.51912470488173
O	0.99347282427381	2.26672978955446	-1.99708869175474
N	-2.86196227237765	0.54066216356201	0.64128434284455
N	2.86195558045350	0.54178460455903	-0.64201038569451
N	-1.55689467503653	-1.54102248182490	-0.53711475761229
N	1.55717890355735	-1.53935269369056	0.53694974086032
O	-0.53157039948203	-2.58062865784802	3.20795240972728
O	0.53438843525241	-2.58285398442219	-3.20542964439986
C	-3.60750964630460	0.80177931620085	-0.61008393533339
C	-3.14824141619581	2.06490322916993	-1.29622195275629
C	-3.24678259059186	1.53132421278689	1.67117640855038
C	-2.16135498029843	1.76275760423727	2.68718903573668
C	-0.02014517699981	2.70719557939379	2.95901456493709
C	1.22376238216791	3.12338948579111	2.23409450180599
C	0.01933836290771	2.70888252577312	-2.95853665311406
C	-1.22445505591904	3.12419051579664	-2.23315898925725
C	3.24637052087995	1.53263083534645	-1.67196014651928
C	2.16060692593966	1.76412850548512	-2.68769225394163
C	3.14777403868059	2.06571851588635	1.29560638250347
C	3.60755069012719	0.80284446454373	0.60932038167673
C	-3.16303291291167	-0.81998307867341	1.12844950480508
C	3.16312692617585	-0.81873632963789	-1.12926348927474
C	2.61462163719562	-1.88059815425226	-0.21520438506174
C	-2.61459250518194	-1.88202861206323	0.21461915306928
C	1.06544087373623	-2.41380865592239	1.43444240000034
C	1.56478307698164	-3.70595149582365	1.55914125760106
C	2.63069712291857	-4.08557125209501	0.74275358867276
C	3.18066105698317	-3.15606441492244	-0.13577400386211
C	-3.18030325710795	-3.15767013470100	0.13560208972293
C	-2.63014130853059	-4.08727436304834	-0.74270010765632
C	-1.56382962918483	-3.70785724493783	-1.55861469962513
C	-1.06472498224223	-2.41563850633231	-1.43404616673736
H	-4.25223901897023	-0.96526168390170	1.24946997086011
H	-2.69951971647908	-0.93532524552212	2.11849937887087
H	4.25230638900830	-0.96406128444765	-1.25057553385063
H	2.69935233080401	-0.93408570813650	-2.11919429882410
H	1.12915656880639	-4.38121146117287	2.29281614974618
H	3.04833638477133	-5.08949239884034	0.81127699308985
H	4.04439930210720	-3.40608145578298	-0.75045358494329
H	-4.04375073174798	-3.40784348075511	0.75062116328168
H	-3.04745230588191	-5.09135920018889	-0.81089292975271
H	-1.12806079023469	-4.38308759582212	-2.29217151228018
C	-0.02749715156857	-1.85701868266623	2.32845251841396
C	0.02806162949364	-1.85848429468307	-2.32786386839124

H	-4.69423906236454	0.87554710015195	-0.42059700897638
H	-3.66016964124749	2.15731056775581	-2.26603670683747
H	-3.36424230487729	2.97232743869459	-0.71156262694937
H	4.69423046992054	0.87712042666857	0.41973840868590
H	3.44657648615733	-0.04376403153948	1.28672384274692
H	3.66018274556886	2.15861529318870	2.26511928291878
H	3.36280180712589	2.97323056975011	0.71071379347308
H	1.88035368106306	0.84478429329947	-3.22271899524562
H	2.49823821479419	2.51732041193359	-3.41604217656192
H	3.46027833432880	2.48665476605078	-1.17814050739079
H	4.17161450887745	1.22341025681282	-2.18963348200676
H	-2.49940586410864	2.51560580894174	3.41569953350432
H	-1.88095782673393	0.84333070673027	3.22201939543369
H	-4.17204734780490	1.22186235075845	2.18866712804113
H	-3.46082105341025	2.48539189722717	1.17749662516704
H	-1.03329396094650	3.94508723728727	-1.52325518250411
H	-1.97044377318818	3.45993505372389	-2.96761901841406
H	-0.20402870231444	1.88382796625309	-3.65456375590469
H	0.42142138655560	3.56107850566123	-3.52893437620078
H	-3.44612370109985	-0.04462061948638	-1.28763293580224
H	1.96952260094930	3.45860108812877	2.96903956252027
H	1.03264264184275	3.94486748653184	1.52484391776883
H	-0.42227054616203	3.55880464832219	3.53026099124938
H	0.20321254753706	1.88146048937511	3.65423403243513

La(H_2O)Macropa$^+$

La	12.13548765522184	8.59142284057583	5.47102883290005
O	14.94007291629478	8.70510288519109	5.37648707727339
O	9.98326866583254	9.90345986615250	4.24367504129680
H	13.37127626933476	10.14544559417767	2.90633178697739
O	13.58756067594259	7.84139886930426	3.04359174150209
O	12.87570636384187	6.25795447405030	5.32018071965907
O	9.28192689258227	8.42558797727479	9.01726375800578
O	10.57552880052442	9.08395346997461	7.29202931620839
N	10.04890883945065	6.92597110056418	5.89131721098707
O	12.91630256197164	4.14785786568175	6.11336049755494
N	13.59106312008390	9.95485341572376	7.63685471589947
N	12.90445892674220	7.31075017275595	7.72584670088013
C	9.43688833511039	7.96306509479620	2.96439085291101
H	8.71440796869285	7.71297725549376	3.75039965289818
H	9.01342901802358	7.61815250896012	2.00353792196884
N	10.70892993293604	7.26063830415834	3.25647624777809
C	12.95573167914273	6.81773741117531	2.25615143694783
H	13.04697652927110	5.85672631507905	2.78532918953940
H	13.47267793185472	6.73301807914078	1.28652665873956
C	13.11239126446499	7.19316960204666	10.10405896096004
H	13.22025146602659	7.71547225645895	11.05384467609559

C	15.60408580731026	8.56759037532468	4.10918025169032
H	15.56677965452398	9.52643757197044	3.56546114540730
C	12.98444486242407	5.17314068944592	8.80791133938461
H	12.98142556446849	4.08962336316180	8.70794991139822
C	14.94773191803374	7.47111851569959	3.32470525248940
H	15.49640230922977	7.32465319920996	2.38012106332453
H	14.95113433806586	6.52833694669003	3.89467094450770
C	15.58830054832079	9.71311445628643	6.17116402624002
C	15.03803724285136	9.64040469710013	7.57153587809673
H	15.18797783577155	8.61981104034544	7.94334179759786
C	12.91701917783043	5.96654327029214	7.66639789390980
C	9.78979503141358	8.26197401050343	7.88949019417456
C	8.86973807201937	4.88829734674574	5.47517945081858
H	8.63387221895187	4.07568940106878	4.78919097865187
C	11.51813667372827	7.20230506265259	2.01930954316354
H	11.49482787081762	8.19402277464459	1.54956019693679
H	11.07282619924225	6.49127908277026	1.29857659802078
C	12.89556975293803	5.38639459969069	6.26340504816293
C	10.39969577540865	5.89419198665856	3.72200585277904
H	11.34074727151129	5.33390202608573	3.80168149464648
H	9.75020148692189	5.36677247955904	2.99932467579380
C	13.04800371772207	9.42694757773848	8.90426360268934
H	13.62963831761024	9.79630874082321	9.76885107523477
H	12.01570769663744	9.78490024228871	9.01469212496070
C	9.74708618985562	5.90137853102642	5.07791186569351
C	8.59348143439298	6.02805893706394	7.57229457237840
H	8.15351406720282	6.13537444400744	8.56167842143947
C	9.59270578082319	9.46102452798011	2.93226309038805
H	10.34079179866150	9.79861845934162	2.19517176552985
H	8.62675226611757	9.91878796223705	2.66661456365093
C	13.07109318526951	5.80256243374181	10.04922066963916
H	13.12962299591235	5.21459037752178	10.96466377525830
C	13.02188518383397	7.92230652925766	8.91499132326127
C	9.46335751433606	7.00679199745134	7.10059729390654
C	8.30273515485777	4.94360073238574	6.74538193637901
H	7.62378082938543	4.15950515090211	7.07898066263559
O	11.62660392673646	11.39981452638858	5.99186134373395
O	12.97106468130076	10.40030114015192	3.75565339160566
C	9.90777364676553	11.33605999619338	4.32914471535492
C	13.41677084168425	11.42254983654515	7.58287560173705
H	16.66101187897425	8.30539118733035	4.27688951970076
H	15.43837480485660	10.70004224908232	5.70185968543248
H	16.67123606935440	9.51367061643836	6.19930674118365
H	15.61337287614334	10.31970338877542	8.22660643510432
C	10.26397523350560	11.76556279913167	5.72060628959306
C	11.99909688638545	11.85084701499839	7.30501192264111
H	12.91405820936679	11.36959893452260	3.78758865580213
H	8.87977120484289	11.66250687019804	4.10348013435640

H	10.58517300147065	11.78823705340411	3.58528534362340
H	13.75187700427956	11.88802049343739	8.52861425914653
H	14.06091615253790	11.81312073275498	6.78469556780073
H	9.60697045843992	11.27819828597390	6.45881456315797
H	10.14885831233637	12.85884767301862	5.80129194397539
H	11.28864742363407	11.43834079518551	8.03809578959177
H	11.93962482963714	12.95077950806008	7.34237181923581

La(OH)Macropa

La	12.20998551095127	8.62070980254255	5.38042995731878
O	15.04847821965605	8.56998866847044	5.40155538250155
O	9.90688346013559	9.91719704685778	4.29424883088850
H	11.86383001297195	12.97365110593348	7.48989983202683
O	13.63814608235895	7.69416401617680	3.03645320935370
O	12.88157223608905	6.17961041075983	5.31309689275510
O	9.29971783270650	8.41057667838715	8.99732432182054
O	10.61521017053484	9.08108969416893	7.29246246840564
N	10.01859822610306	6.95855958133856	5.83264422129549
O	12.88500310605236	4.10334157739689	6.19346040430785
N	13.60345479256289	10.00383131012704	7.60121494867887
N	12.98458193512406	7.32173549536625	7.70755050178586
C	9.50632240673585	8.02271212773979	2.88214675395380
H	8.73504143774201	7.76001413602418	3.61658819423982
H	9.12145218299090	7.73624883223933	1.88576946884264
N	10.73680223974121	7.26118121220566	3.20068876052809
C	12.98329105506941	6.68963694585289	2.25874439580848
H	13.00881004662248	5.73048546024626	2.80036675143752
H	13.51106976852607	6.55954744838979	1.29811546847731
C	13.18397410096554	7.23881343419786	10.09113018435434
H	13.29417274239621	7.77672430110018	11.03240244755605
C	15.62797439366941	8.46822648848599	4.09001096969641
H	15.46357198080404	9.41493112298362	3.55021114192929
C	13.01906233959846	5.20216078548781	8.83022162682493
H	12.98914699093920	4.11748210476611	8.74733484226334
C	14.98915381263777	7.33273220300719	3.34032251783572
H	15.54970526932846	7.15945989900086	2.40551228266023
H	14.99862210759112	6.40717941019662	3.94016413367006
C	15.56614220564206	9.72815477822986	6.07679486844612
C	15.05715476198464	9.72974166262135	7.49729757953914
H	15.25479469973610	8.74069028998187	7.92890978993405
C	12.96338322280772	5.97718574336551	7.67338360957409
C	9.81371750722569	8.26884901215284	7.86143269379976
C	8.80811551928726	4.93801473791298	5.41192217033517
H	8.55742704734030	4.13226493656623	4.72241353506524
C	11.57158854763242	7.15483672725666	1.98563282613906
H	11.62018903144838	8.14865283583258	1.52166577756836
H	11.10235814520261	6.47106918020533	1.25176460480107

C	12.89948358066207	5.35454912405128	6.28567694132698
C	10.36528828058835	5.91479634648728	3.66945824618696
H	11.28400334906342	5.32044481397796	3.76769996309875
H	9.70842857352400	5.40485352507242	2.93925151538027
C	13.10531782903371	9.45326324955883	8.87277563567909
H	13.69451095091579	9.83210440646573	9.72978424314728
H	12.06617943598699	9.78288051353995	9.00695132824411
C	9.69974807844710	5.94161224051775	5.01942862749108
C	8.56859738440277	6.06376719318246	7.52024278666639
H	8.14211244022375	6.16773153733676	8.51611663349588
C	9.69933170234909	9.51983252042261	2.92849935538774
H	10.55868035263065	9.85314923288081	2.32129316575061
H	8.79174334768998	10.01002120412497	2.53920497770459
C	13.12608232607067	5.84841980648287	10.06074243701529
H	13.17913372662831	5.27465678647225	10.98593708647682
C	13.10098580440975	7.94773159600456	8.88818655120551
C	9.45115800868207	7.03404824851278	7.04916846929872
C	8.24922531581786	4.99340001921339	6.68582915693929
H	7.55801541335118	4.21877826588143	7.01777281730740
O	11.52596218268606	11.49090288302337	6.06980032859473
O	13.00563169641605	10.30258752547324	4.02130743638670
C	9.82037512114605	11.34580447777101	4.41737985695577
C	13.38448962252596	11.46637358080642	7.56887682634795
H	16.71182708645501	8.28572296936450	4.18371669676843
H	15.25801093379119	10.62991580578549	5.52097634068303
H	16.66810858598163	9.68202358951136	6.08642544261471
H	15.63160690820470	10.46561549264887	8.09013419403797
C	10.14129182448908	11.75832697137148	5.82842874135232
C	11.94047052478764	11.87800120920129	7.38276321343568
H	12.68212990774134	11.12197985261606	4.43669260991985
H	8.79795426461929	11.67249555343456	4.16376866572175
H	10.52397770780008	11.82078214177422	3.71180202068970
H	13.76722469163277	11.93228004503382	8.49793664300698
H	13.97085049339744	11.87577697608347	6.73552596261550
H	9.52920245416580	11.19555357572609	6.55164194167354
H	9.93751936114396	12.83681894081649	5.94219266933377
H	11.27593258362640	11.40839657579717	8.12464910564008

LaFMacropa

La	2.38125701045825	4.12665783279925	10.65267883448133
O	3.00081389042236	5.41449941088905	8.57037142644427
O	3.84661104298963	7.22435719592947	7.52428353031277
O	-0.01262265553173	4.47790134129261	9.22062877418849
O	2.02768173563235	2.81250750997104	8.04779906089515
F	1.25881918791056	2.18894005259087	10.65350968114803
N	1.94355670027367	6.87325787473815	10.50837329239322
N	4.68854031667931	2.83833532507478	9.28360377861373

C	3.21571800648985	6.66632568277778	8.45386062021862
C	2.61960270882367	7.53459070219708	9.55180542829571
C	2.74256368257216	8.92250281819813	9.54944991635118
H	3.30874238231360	9.40662121598595	8.75609652281137
C	2.11873540809996	9.64808286168068	10.56316621479723
H	2.18778537342178	10.73546644604570	10.59027005416199
C	7.06982279278101	5.68246314472864	9.77691270584649
H	7.79615813986473	5.49559110182604	8.98636227559181
C	5.88875961463836	4.93491453987684	9.82955833134821
C	5.57398016290288	3.90227957338447	8.78126221425586
H	5.05748229505825	4.41629659221915	7.95917636870775
H	6.51526632120292	3.48112151208101	8.37915569092327
C	5.42700132444448	2.01139384724844	10.26615217892823
H	6.08188344873776	1.28506202994534	9.74983944012405
H	-0.73099318819942	6.59288788256975	10.44819805012371
C	-1.15699587340931	4.54520615540602	10.08887065760260
H	-1.26984398432618	3.58205704347625	10.61549513179121
H	-2.06318682332372	4.71335661798633	9.48396398928392
C	-0.24019060520640	3.52198418877431	8.16944303676481
H	-1.10126278779257	3.84952569184668	7.56353353873016
H	-0.46600017968671	2.53896057988166	8.61408964242586
C	0.98026338196072	3.42759881384482	7.29570514363413
H	0.73833376863638	2.81299068146983	6.41101068839520
H	1.30135114896787	4.42776562081169	6.95883003113289
C	3.21258887453980	2.63218707538805	7.27147899720513
H	3.54760576917279	3.60500786773693	6.87685186220305
H	3.00295128322854	1.96467662845982	6.41751269411682
C	4.25239790019587	1.98469943584743	8.15781609134627
H	3.82236311706927	1.06570108964589	8.57684527294588
H	5.12134496263998	1.68881110624851	7.53854356744288
O	1.42121545598878	3.16331587474315	13.25877911476409
N	0.11210209809932	5.48005575015267	12.02285048880257
O	3.88075761313876	2.22881943771810	12.08238008441489
O	3.18560690138581	5.30720432964406	12.73386429336671
N	4.97886773982576	5.12090607902408	10.79722826136496
C	1.32662699027030	7.56864958025508	11.47523457184090
C	1.38598039944477	8.96504732276258	11.52961297452808
C	7.29628223580559	6.65628752019352	10.74535221825258
C	4.51899483645555	1.27236844274559	11.21900941027946
H	6.07445315980318	2.66998404457471	10.85806976251656
C	-0.97451339406895	5.70238454574891	11.04074867944942
C	2.47859800356020	2.56395054891039	14.00936596204602
C	0.67529758866462	4.10100915259717	14.03543547933079
C	-0.40714068350669	4.67671261657580	13.15053041567495
C	0.58838070223968	6.78040950547244	12.52298118398596
C	3.16912482135704	1.55369892507019	13.13509700974455
C	4.16544620605735	6.11470194252450	12.85461904305327
C	5.21552752314756	6.03344750285438	11.75665999308254

H	0.86032129784224	9.50056477894571	12.31977823123569
C	6.35778101359546	6.83143017774657	11.76116401538404
H	8.20478084252756	7.25790405599652	10.71972081655370
H	3.74648267262090	0.68523494271448	10.69337632905642
H	5.12144547377876	0.57816505731801	11.82768858121920
H	-1.93119492993016	5.90382107340009	11.55757084193867
H	2.06881003373372	2.04778845536429	14.89516406805234
H	3.18516583280428	3.34208598708234	14.34446430651515
H	0.20379461170096	3.58744353925601	14.89144962759477
H	1.35184404114437	4.87772797113665	14.42697143633072
H	-0.98863602584798	3.84422547813349	12.73348496976667
H	-1.09686111638507	5.28200892654491	13.77018544868325
H	-0.24815443609051	7.38475246294766	12.92301681408159
H	1.29077663080476	6.59243554345998	13.34647908391560
H	2.43043432846119	0.86545445023160	12.69257842457495
H	3.88498377875296	0.97299293976086	13.74021774937030
O	4.33651303191057	6.93465016208136	13.78844475619429
H	6.49605906625461	7.56082478543767	12.55689681705098

2.3 X-ray crystallography

Table S4. X-ray crystallographic data collection and refinement parameters for complexes **5**, **6** and **8**.

	[LaF(macropa)] (**5**)	2[La(H_2O)(macropa)]OTf (**6**)	[LaF(DOTpy)]OTf$_2$ (**8**)
Chemical formula	$C_{26}H_{34}FLaN_4O_8$	$C_{60}H_{101}ClF_6La_2N_9O_{31}S_2$	$C_{34}H_{40}F_7LaN_8O_6S_2$
Formula weight	688.48	1935.89	992.77
Space group symbol	P 31 2 1	P 21	P 21 21 2
a, Å	15.4947(1)	16.7514(3)	22.1793(2)
b, Å	15.4947(1)	10.1970(2)	11.1061(1)
c, Å	10.6535(1)	23.4460(5)	8.0043(1)
α, deg	90	90	90
β, deg	90	96.158(2)	90
γ, deg	120	90	90
V, Å3	2215.08(4)	3981.80(14)	1971.66(4)
Z	3	2	2
R(reflections)	0.0249(2925)	0.0186(6237)	0.0402(3873)
wR2(reflections)	0.0645(2953)	0.0488(6375)	0.1005(3946)
GOF(S)	1.083	1.041	1.081

Table S5. La−X bond length (Å) for complexes 5, 6 and 8.

	[La**F**(macropa)] (**5**)	[La(H_2O)(macropa)]OTf (**6**)	[La**F**(DOTpy)]OTf$_2$ (**8**)

La−X bond length, Å	2.286(5)	2.4882	2.256(5)

3 References:

1. L. S. Natrajan, N. M. Khoabane, B. L. Dadds, C. A. Muryn, R. G. Pritchard, S. L. Heath, A. M. Kenwright, I. Kuprov and S. Faulkner, Inorganic Chemistry, 2010, 49, 7700–7709.
